# Light-induced changes of far-red excited chlorophyll fluorescence: further evidence for variable fluorescence of photosystem I *in vivo*

**DOI:** 10.1007/s11120-022-00994-9

**Published:** 2023-01-04

**Authors:** Ulrich Schreiber

**Affiliations:** Julius-von-Sachs Institut für Biowissenschaften, Universität Würzburg, Julius-von-Sachs Platz 2, 97082 Würzburg, Germany

**Keywords:** *Chlorella*, Room temperature fluorescence emission, Far-red excited chlorophyll fluorescence, F > 765 nm, Fv(I), MULTI-COLOR-PAM, Polyphasic fluorescence rise O-I_1_-I_2_-P, Far-red absorption, “Red Chls”

## Abstract

Recently, the long-standing paradigm of variable chlorophyll (Chl) fluorescence (Fv) *in vivo* originating exclusively from PSII was challenged, based on measurements with green algae and cyanobacteria (Schreiber and Klughammer 2021, PRES 149, 213-231). Fv(I) was identified by comparing light-induced changes of Fv > 700 nm and Fv < 710 nm. The Fv(I) induced by strong light was about 1.5 × larger in Fv > 700 nm compared to Fv < 710 nm. In the present communication, concentrating on the model green alga *Chlorella vulgaris*, this work is extended by comparing the light-induced changes of long-wavelength fluorescence (> 765 nm) that is excited by either far-red light (720 nm, mostly absorbed in PSI) or visible light (540 nm, absorbed by PSI and PSII). Polyphasic rise curves of Fv induced by saturating 540 nm light are measured, which after normalization of the initial O-I_1_ rises, assumed to reflect Fv(II), display a 2 × higher I_2_-P transient with 720 nm excitation (720ex) compared with 540ex. Analysis of the Fo(I) contributions to Fo(720ex) and Fo(540ex) reveals that also Fo(I)720ex is 2 × higher than Fo(I)540ex, which supports the notion that the whole I_2_-P transient is due to Fv(I). The twofold increase of the excitation ratio of F(I)/F(II) from 680 to 720 nm is much smaller than the eight–tenfold increase of PSI/PSII known from action spectra. It is suggested that the measured F > 765 nm is not representative for the bulk chlorophyll of PSI, but rather reflects a small fraction of far-red absorbing chlorophyll forms (“red Chls”) with particular properties. Based on the same approach (comparison of polyphasic rise curves measured with 720ex and 540ex), the existence of Fv(I) is confirmed in a variety of other photosynthetic organisms (cyanobacteria, moss, fern, higher plant leaves).

**Supplementary Information:**

The online version contains supplementary material available at 10.1007/s11120-022-00994-9.

## Introduction

Since the pioneering work of Duysens and Sweers (1963), a general consensus among photosynthesis researchers has developed that photosynthesis consists of two light reactions, PS I and PS II, that are connected via an intersystem electron transport chain and that variable chlorophyll fluorescence, Fv, *in vivo* is originating from Chl *a* in PS II, acting as an indicator of the efficiency of energy conversion in PS II. Hence, it has been generally assumed that Fv consists exclusively of Fv(II). At the same time, it is well established that fluorescence excited in PS I, F(I), does contribute significantly (up to 50%) to the dark fluorescence yield, Fo, particularly at wavelengths beyond 700 nm (Genty et al. 1990; Pfündel 1998; Franck et al. 2002).

Lazar (2013) put forward theoretical arguments for the existence of Fv(I) based on literature values on F(I) properties of PS I particles, incorporated in a model of energy conversion in PS I. Dusan Lazar predicted characteristic transients of Fv(I) in the sub-s time range upon dark–light induction in strong light, resulting in an 8–14% contribution of Fv(I) to the overall fluorescence rise. The predicted Fv(I) showed as a transient peak at about 100 ms after onset of saturating illumination, which falls into the time range of the I_2_-P transient of the polyphasic fluorescence rise (Schreiber 1986; Neubauer and Schreiber 1987; Schreiber and Neubauer 1987). Based on parallel measurements of P700 and fluorescence, it had previously been shown that the reduction of P700 after closure of the PS I acceptor side does coincide with I_2_-P, leading to the suggestion that I_2_-P may reflect Fv(I) (Schreiber et al. 1989). This suggestion was further substantiated by simultaneous *in vivo* measurements of ferredoxin, P700 and fluorescence upon onset of saturating illumination (Klughammer and Schreiber 2016).

Very recently, more direct evidence for the existence of appreciable amounts of Fv(I) in suspensions of *Chlorella vulgaris* and *Synechococcus leopoliensis* was presented (Schreiber and Klughammer 2021) by comparing the polyphasic rise kinetics of F > 700 nm with F < 710 nm measured in parallel under close to equal irradiation conditions. If the light-induced increase of overall fluorescence yields were due to Fv(II) only, the kinetics of Fv > 700 nm and Fv < 710 nm should be equal. In reality, however, the amplitude of I_2_-P was found higher in Fv > 700 nm compared to Fv < 710 nm by a factor of about 1.5 in *Chlorella* and 2 in *Synechococcus* (Schreiber and Klughammer 2021). The F(I)/F(II) emission ratio is known to increase at wavelengths > 700 nm (Franck et al. 2002; Wientjes et al. 2017). Determination of Fv(I) relied on the generally accepted notion that the rapid initial “photochemical” O-I_1_ transient reflects the closure of PS II reaction centers and, hence, consists of Fv(II) only. After O-I_1_ equalization, with all changes due to Fv(II) being equal, the difference between the F > 700 nm and F < 710 nm kinetics was interpreted to reflect the kinetics of Fv(I), which displayed a transient peak around 150 ms in *Chlorella* and 200 ms in *Synechococcus*, thus confirming the theoretical predictions of Lazar (2013). The “extra Fv(I)” revealed in Fv > 700 nm showed very similar kinetics as the I_2_-P transient, all of which was suppressed by DBMIB, thus suggesting that the “extra Fv(I)” in the F > 700 response actually corresponds to an “extra I_2_-P.” Consequently, it was assumed that the whole I_2_-P transient is due to Fv(I). Based on this assumption, it was possible to deconvolute the overall polyphasic rise kinetics into its Fv(I) and Fv(II) components. In *Chlorella*, deconvolution of Fo(> 700) indicated a 37% Fo(I) contribution, in line with the literature values for C3 photosynthetic organisms (Genty et al. 1990; Pfündel 1998; Peterson et al. 2014), thus supporting the assumption that the whole I_2_-P transient reflects Fv(I). The transient character of Fv(I) was explained by the notion that for the appearance of Fv(I) both the primary acceptor, ferredoxin, and the primary donor, P700, have to be reduced (Schreiber and Klughammer 2021), which is rarely the case under natural physiological conditions: After dark-adaptation P700 is reduced and Fd oxidized, so that upon illumination initially normal charge separation can take place and Fv(I) is photochemically quenched. When during illumination P700 becomes oxidized and Fd reduced, Fv(I) is non-photochemically quenched by P700^+^. As soon as the reactions downstream of PSI become light-activated, Fd is reoxidized and Fv(I) again is photochemically quenched. Hence, the time window during which Fv(I) can be observed is relatively short. In experiments with *Chlorella* it reaches from about 20 ms to 800 ms after onset of strong actinic illumination. In green algae and cyanobacteria, the P-S fluorescence decline, which reflects activation of the reactions downstream of Fd, sets in already within 100–200 ms after onset of illumination (see e.g., Fig. 5 in Schreiber et al. 1995).

The present communication on Fv(I) in *Chlorella* builds upon the preceding study of Schreiber and Klughammer (2021). Again the experiments consist of measurements of the polyphasic fluorescence rise kinetics upon the onset of strong actinic light and again kinetic information on Fv(I) is obtained by the comparison of two closely linked measurements, carried out in parallel under close to equal conditions, both with respect to the physiological state of the sample and the photosynthetically active radiation (PAR) seen by the monitored cells: Far-red pulse-modulated measuring light (ML) (in most experiments peaking at 720 nm, referred to as 720ex), which is more strongly absorbed by PS I compared to PS II, is supposed to preferentially excite F(I), whereas 540 nm pulse-modulated ML (540ex) should excite both photosystems about equally. Hence, if I_2_-P reflects Fv(I), it should be distinctly more pronounced with 720ex compared to 540ex. While this may be considered a relatively straight forward concept, in practice, measuring light-induced changes of far-red (FR) excited chlorophyll fluorescence constitutes a rather difficult task for six major reasons:

1. Light absorption by Chl *a* drops to relatively low values above 700 nm, resulting in correspondingly weak fluorescence signals.
2. Most of this weak FR excited fluorescence, which peaks around 725 nm, is absorbed by the long-pass detector filter (opening above 750 nm), which serves to minimize the fraction of pulse-modulated FR-ML that reaches the detector.
3. The standard red glass filter RG780 (Schott), which as far as its transmission properties are concerned, should qualify as an effective long-pass filter, displays considerable near-infrared fluorescence upon absorption of far-red light.
4. The intensity of the fluorescence that is excited by the continuous actinic illumination is several orders of magnitude larger than the intensity of the fluorescence excited by the FR-ML. Hence, for reliable detection of the latter, a very selective amplifier is required, which allows to monitor small pulse-modulated signals against a vast background of non-modulated signals.
5. As will be revealed by the present study, only part of light-induced changes of FR excited fluorescence reflects the originally expected variable fluorescence of PS I, Fv(I). Furthermore, detection of this part is aggravated by the fact that Fv(I) is transient, in *Chlorella* reaching a peak within 100-200 ms upon strong illumination and disappearing again within less than 1 s.
6. Detection of Fv(I) is possible under *in vivo* conditions only, i.e., in an extremely complex physiological system, controlled by numerous regulatory mechanisms, part of which are still poorly understood.

These problematic aspects may explain why so far no reports on light-induced changes of FR excited chlorophyll fluorescence have appeared in the literature.

As will be outlined below, the technical problems were overcome with the help of a purpose-tailored measuring system, based on the combination of a Multi-Color-PAM fluorometer and a Dual-PAM-100 instrument. Using this system it has become possible to measure the polyphasic rise kinetics with 720ex and 540ex routinely under close to equal conditions. Similarly as previously reported for parallel measurements of F > 700 and F < 710, after normalization of the initial O-I_1_ rises, assumed to reflect Fv(II), consistently a more pronounced I_2_-P transient is observed with 720ex compared to 540ex, thus confirming that the “extra I_2_-P” may be considered an “extra Fv(I).” While upon excitation with visible ML (540 nm and 680 nm) close to equal polyphasic rise kinetics were obtained after O-I_1_ normalization (including equal amplitude I_2_-P transients), a selective increase of the I_2_-P phase was observed in the “red drop” wavelength range at > 680 nm, peaking at about 720 nm. It will be shown that the amplitude of the “extra Fv(I)” in the 720ex response is distinctly smaller than expected in view of PSI and PSII action spectra (Schreiber and Vidaver 1974; Laisk et al. 2014). The same also holds for the Fo(I) contained in Fo(720ex). In both cases the apparent F(I)/F(II) is just by a factor of 2 higher with 720ex compared to 540ex, whereas from the action spectra a factor of 8–10 would seem appropriate. While this apparent discrepancy cannot be resolved conclusively in the present communication, tentative explanations will be presented that emphasize the potential role of the so-called “red Chls” (reviewed in Gobets and van Grondelle 2001; Gobets et al. 2001; Croce and van Amerongen 2013; Krüger et al. 2014; Santabarbara et al. 2020) in determining the properties of Chl fluorescence excited with FR and measured in the near-infrared (F > 765 nm).

## Materials and methods

### Experimental set-up

**Fig. 1:**
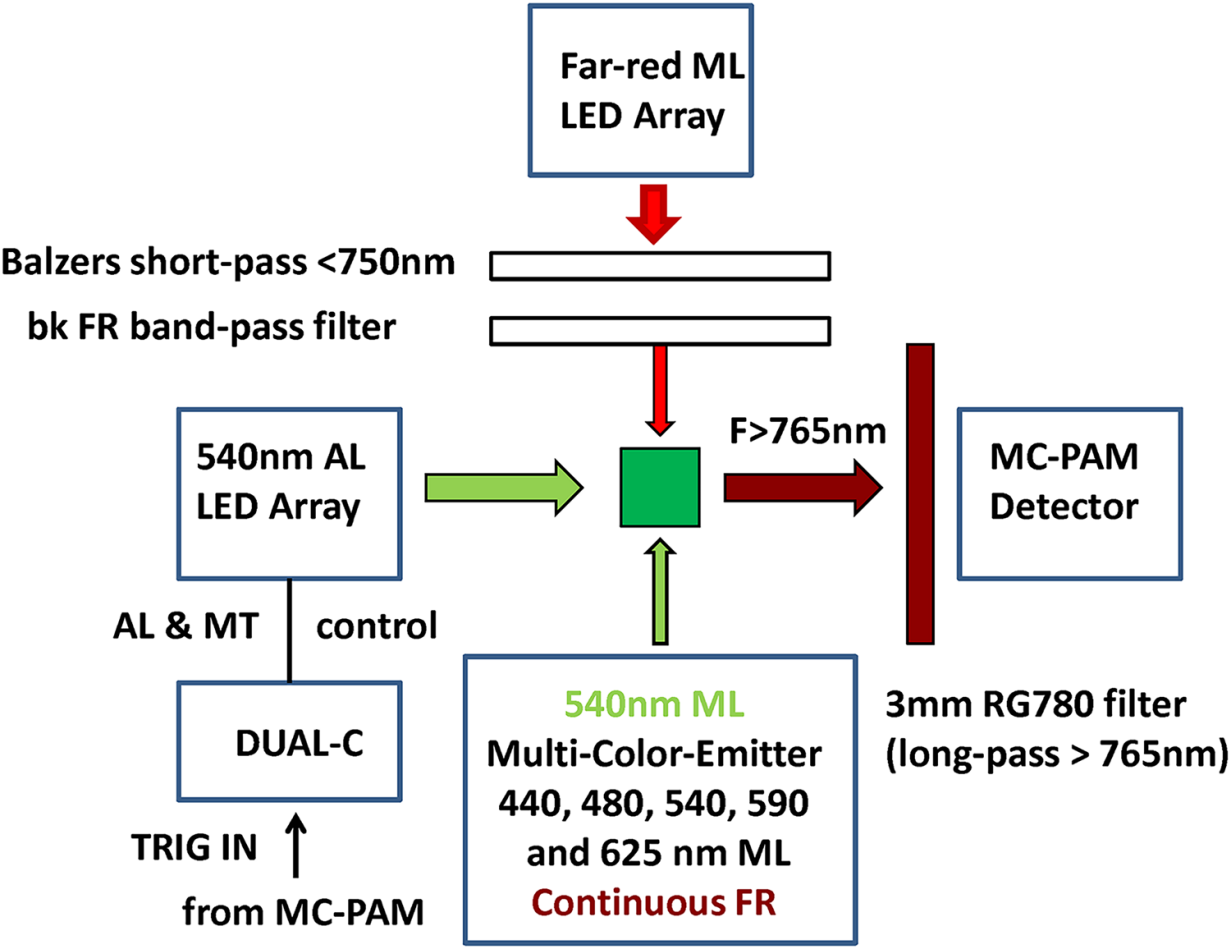
Block diagram of experimental set-up for comparative measurements of light-induced changes of chlorophyll fluorescence yield using far-red (FR) (preferentially 720 nm) or visible (preferentially 540 nm) pulse-modulated excitation. The optical geometry is optimized for homogeneous illumination by both the two types of measuring light (ML) as well as the 540 nm actinic light (AL) and multiple turnover flashes (MT). The relative yield of pulse-modulated fluorescence is measured with a Multi-Color-PAM fluorometer (MC-PAM) controlled by the PamWin-3 software. A custom 540 nm LED array is powered by the control unit (DUAL-C) of a DUAL-PAM-100 and controlled via pre-programmed trigger signals obtained from the MC-PAM. For further explanations, see text

The experiments were carried out with an extended version of a Multi-Color-PAM Chlorophyll Fluorometer developed by Christof Klughammer and Ulrich Schreiber (commercially available via Heinz Walz GmbH, Germany). Technical features of this fluorometer were previously described in detail (Schreiber et al. 2012; Schreiber and Klughammer 2021). This instrument is particularly well suited for measuring rapid fluorescence changes in suspensions of algae and cyanobacteria, with variation of the wavelengths of excitation and emission, as well as of the colors of the actinic light driving the changes of fluorescence yield. It combines high sensitivity with high time resolution. The standard version provides pulse-modulated measuring light (ML) at 400, 440, 480, 540, 590, and 625 nm. For the purpose of the present study, the 400 nm excitation was replaced by FR excitation, emitted from a specially designed external FR-LED array, driven by the same current pulses that drive 400 nm ML in the standard version. The two emitter units were mounted at right angles to the Multi-Color-PAM detector in an optical unit with four optical ports (ED-101US/MD, Walz). Figure 1 shows the set-up in a block diagram.

**Fig. 2:**
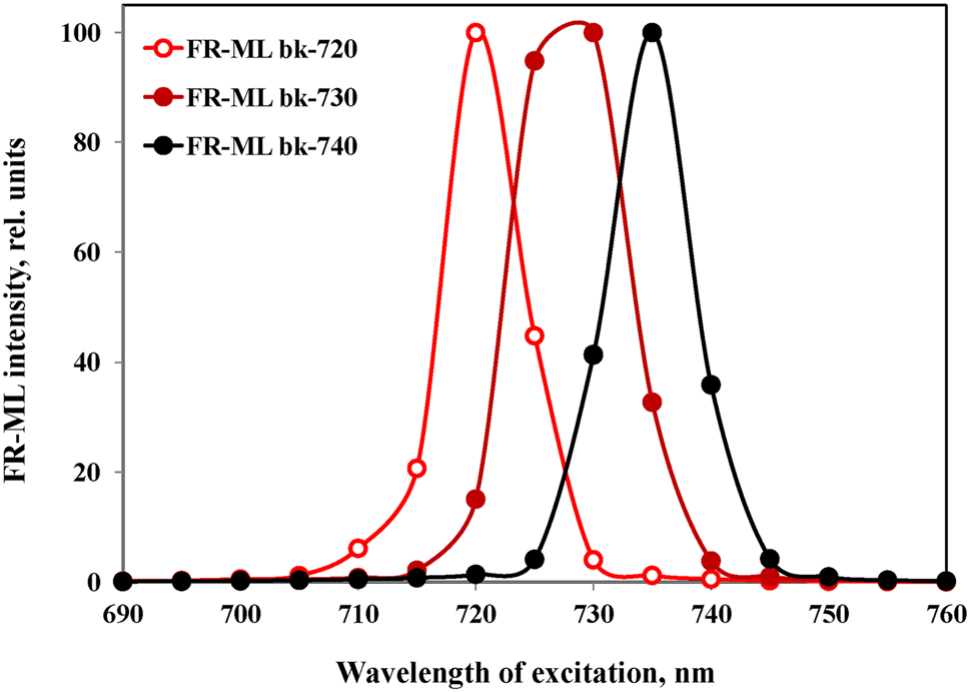
Spectra of three types of far-red modulated measuring light (FR-ML) obtained from a custom FR-LED-Array in combination with different FR bandpass filters (10 nm half-bandwidth, bk Interferenzoptik) and additional short-pass filters (SP750, Balzers) for the elimination of wavelengths > 750 nm. The FR-ML was applied at right angle to the photodiode detector, which was protected by a long-pass filter > 765 nm (3 mm RG780, Schott) (for transmission spectrum, see Fig. 3)

**Fig. 3:**
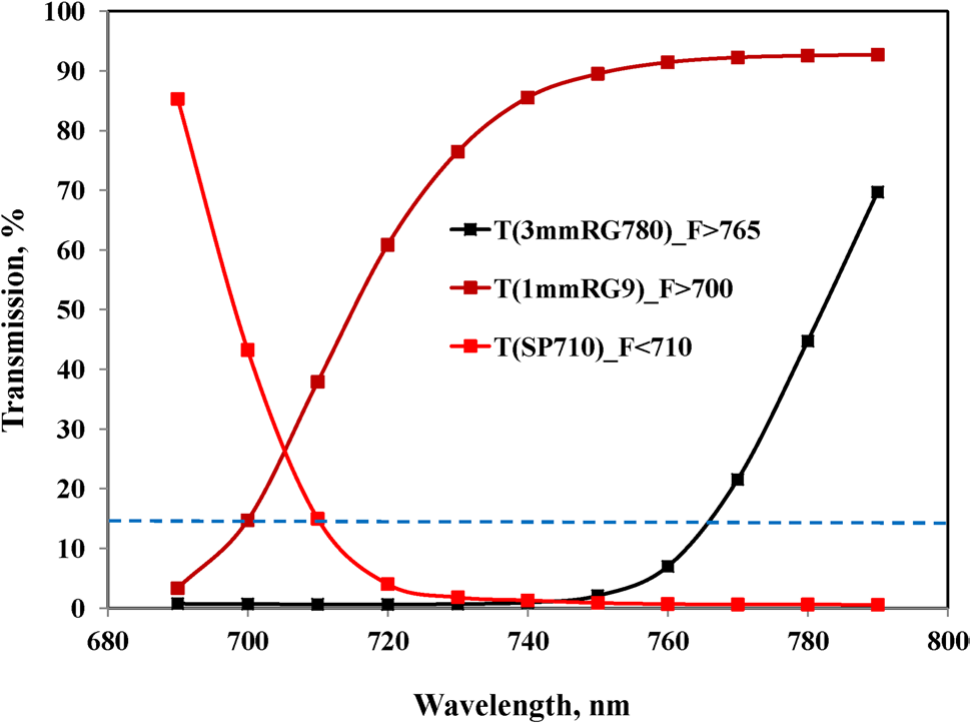
Filters applied in front of MC-Detector for measuring relative fluorescence yield upon excitation with FR or visible pulse-modulated light. The abbreviations for the measured fluorescence (F > 765, F > 700, and F < 710) characterize the wavelengths at which each of the applied filters displays 15% transmission (broken blue line). While F < 710 is enriched in fluorescence originating from PS II, F(II), F > 700, and F > 765 are enriched in fluorescence originating from photosystem I, F(I)

The FR-ML passes a 750 nm short-pass filter (Balzers). An additional bandpass filter with 10 nm half-bandwidth was applied, in most experiments with peak transmission at 720 nm (bk Interferenztechnik, Nabburg, Germany) (see Fig. 2 below for resulting spectra of FR-ML). On the one hand this filter effectively suppresses the short-wavelength tail of the FR-LED emission, which otherwise would cause substantial PS II excitation; on the other hand it cuts off the long-wavelength tail of the FR-LED emission, which overlaps with fluorescence emission. The Multi-Color-PAM (MC-PAM) photodiode detector was protected by a 3 mm RG780 red glass filter (Schott) which blocks transmission below 750 nm (see Fig. 3 below). While most RG780 filters tested by the author displayed relatively high glass fluorescence upon absorption of FR-ML, for the present study a filter was selected with negligibly low fluorescence yield.

A separate custom-made 540 nm LED array was mounted at right angle to the ML sources for providing actinic illumination, i.e., continuous actinic light (AL), strong multiple turnover light pulse (MT), and single turnover flashes (ST). In the case of ST, for obtaining saturating single turnover flashes, in addition to the external 540 nm ST also the internal multi-color ST of the standard MC-Emitter was applied. The 540 nm AL LED Array was powered via the control unit of a DUAL-PAM-100 fluorometer (DUAL-C, Walz) and controlled via pre-programmed trigger signals obtained from the MC-PAM. For this purpose, the TRIGGER OUT BNC socket of the MC-PAM was connected with the TRIGGER IN BNC socket of the DUAL-C and in the Dual-PAM user software Start External Triggering was enabled. Continuous FR for preoxidation of the PQ pool and establishment of state 1 was obtained from the standard MC-Emitter.

The chosen optical geometry assured maximal homogeneity of illumination, so that the FR and 540 nm excited pulse-modulated fluorescence seen by the detector was originating from cells exposed to the same mean 540 nm actinic light intensity. This aspect is particularly important in the context of the present study, in which even small differences between the responses measured with FR and 540 nm ML are analyzed. Both FR and 540 nm light are weakly absorbed by the algal suspension.

The custom-made FR-ML source consisted of a 3 × 3 array of LEDs peaking at 730 nm (Würth Elektronik, type WL-SMDC Horticulture SMT Ceramic 3535) with 30 nm half-bandwidth, focused on a 10 × 10 mm perspex rod which served for homogenizing the FR-ML and for guiding it to the cuvette. Between perspex rod and cuvette a short-pass filter < 750 nm (Balzers) as well as a 10 nm bandpass filter (bk Interferenzoptik) were mounted, with the latter serving for defining relatively narrow bands of ML within the 680–740 nm range (see below). The short-pass filter was essential for lowering the background signal due to stray FR-ML that enters the detector pathway in spite of the 90° geometry and passes the 3 mm RG780 filter in front of the detector.

**Fig. 4:**
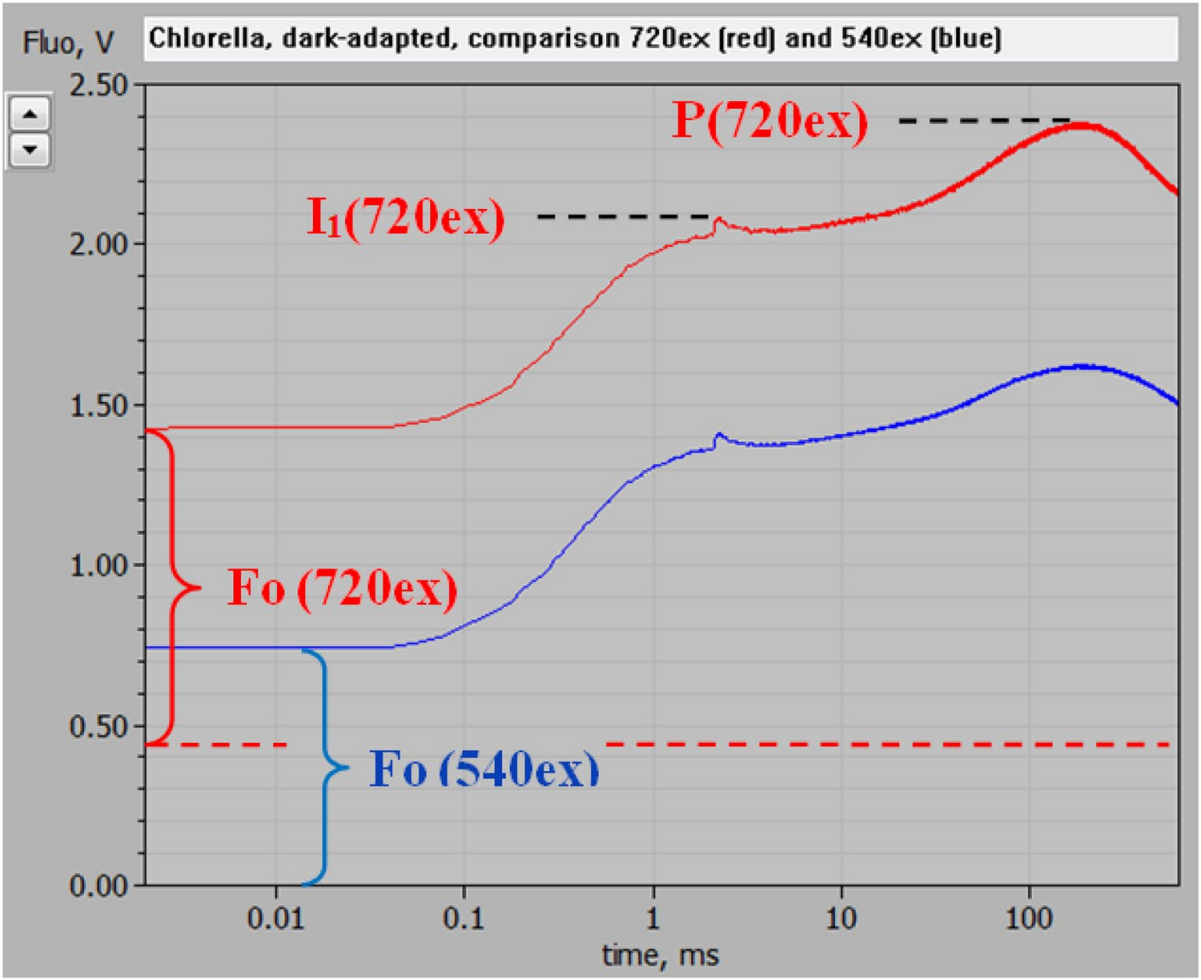
Polyphasic fluorescence rise upon onset of strong actinic illumination measured with dark-adapted *Chlorella* using 720 nm (red trace, 720ex) and 540 nm (blue trace, 540ex) pulse-modulated measuring light. Screenshot of original recordings with PamWin-3 user surface. At 2 ms, i.e., at the end of the initial “photochemical phase,” a saturating single turnover flash was applied for full closure of PS II reaction centers and definition of the I_1_ level. In the given dark state an unequivocal identification of the I_2_ level is not possible (see text). The background signal level with 720ex, as determined under supplementary figures S3–S5, is indicated by the broken red line which thus represents the baseline for the 720ex response, for which the characteristic fluorescence levels Fo, I_1_, and P are indicated. Measurements were carried out in the optical geometry shown in Fig. 1. Actinic illumination with 600 ms Multiple Turnover pulse (MT) of 540 nm light at 8000 µmol m^−2^ s^−1^. Averages of 4 recordings each measured alternatingly using 720ex and 540ex with 5-min dark intervals in between

While with 540 nm ML the background signal was negligibly low, so that Fo could be reliably determined, in the case of FR-ML determination of Fo was complicated by a relatively large background signal. An analysis of this background signal for 720 nm ML is presented under supplementary figures S3–S5. It consists of optical and non-optical components. Whereas the latter could be readily measured (photodiode detector blocked), quantitative determination of the former proved difficult due to its heterogeneity. One part of the optical background signal was due to 720 nm ML reflected from the cuvette walls and another part to 720 nm ML scattered by the *Chlorella* cells towards the detector. The reflectance part can be estimated by measuring the “blank” signal (cuvette filled with suspension medium). For the estimation of the scattering part, first the concentration of freshly precipitated BaSO_4_ was determined that gave the same scattering signal as the *Chlorella* suspension (without RG780 in front of the detector) and then the apparent fluorescence signal of this BaSO_4_ suspension was measured (with RG780 in front of the detector). In this way, the overall background signal with 720 nm ML could be estimated and consequently also an estimate of the Fo could be provided (see Fig. 4 below). This estimate relies on the assumption of equal optical background signals with the used BaSO_4_ and *Chlorella* suspensions, which appears plausible in view of the fact that we are dealing with the reflection and scattering of wavelengths > 765 nm, which should not be affected by the color of the *Chlorella* cells.

A set of 10 nm bandpass filters peaking at 680, 690, 695, 700, 710, 720, 730, and 740 nm was used for investigating the stimulation of Fv(I) in the so-called red drop region of photosynthesis, where PS II action drops steeply at wavelengths > 680 nm, whereas PS I action first further rises to a peak around 685 nm, before it also drops above 690 nm (see supplementary figures S1, S2). For optimal measurements of Fv(I) it is important that the PS I/PS II excitation ratio is maximal, which requires thorough suppression of any tail of wavelengths < 700 in the FR-ML. Figure 2 shows the spectra of the pulse-modulated FR-ML obtained with the bk-720, bk-730, and bk-740 bandpass filters.

The FR-ML which is obtained using the bk-720, bk-730, and bk-740 bandpass filters for excitation of chlorophyll fluorescence is practically free of wavelengths < 700 nm, so that PS II excitation is minimized. In the following text, the abbreviated terms 720ex, 730ex, 740ex, etc. are used for excitation with particular wavelength bands of pulse-modulated ML. In most experiments, 720ex is used for measurements with preferential excitation of PS I.

Opposite to the FR-ML source the Multi-Color-Emitter unit was mounted which provided 540 nm pulse-modulated ML as well as continuous AL, saturating single turnover flashes (ST), and continuous FR. The 540 nm ML (540ex) is similarly to 720ex weakly absorbed and, hence, excites fluorescence homogeneously throughout the sample. Analogously, also the 540 nm actinic light (AL, MT and ST), which is applied at right angles to the ML source, is homogeneously absorbed, thus eliciting equal physiological reactions throughout the sample, which are monitored with 720ex and 540ex under close to identical conditions.

The MC-PAM photodiode detector was protected by 3 mm red glass filter RG780 (Schott), selected for negligibly low fluorescence upon absorption of 720 nm ML. This filter opened at about 750 nm, displaying about 15% transmission at 765 nm. Hence, in analogy to the filter sets used in our preceding study on Fv(I), where F > 700 and F < 710 were compared (Schreiber and Klughammer 2021), the fluorescence measured with 3 mm RG780 is referred to as F > 765 (see Fig. 3 for transmission spectra).

### PAM measurement and the light-driven polyphasic rise of fluorescence yield

The Multi-Color-PAM fluorometer used in the present study, like all PAM devices applies pulse-modulated ML and a special window-amplifier that is selective for the fluorescence excited by individual µs pulses of ML, so that the measurement of the ML-excited fluorescence is not disturbed by the fluorescence excited by much stronger AL or MT (Schreiber 1986). Hence, as ML intensity is constant during measurements, the ML-excited fluorescence may be considered a measure of relative fluorescence *yield* that varies between a minimal value of Fo (dark-adapted sample, primary acceptor Q_A_ fully oxidized) and Fm (Q_A_ fully reduced in the absence of non-photochemical quenching). In contrast, fluorescence *intensity* may vary indefinitely, depending on the intensity of the applied non-modulated actinic illumination. The output of the Multi-Color-PAM is a voltage signal that can vary between 0 and 6 Volt. The amplitude of this signal not only depends on the relative fluorescence yield of the sample, but also on chlorophyll content, the ML color, the chosen settings of ML intensity, and amplifier gain as well as on the choice of optical detector filters. Hence, while the signals are always proportional to fluorescence yield, the units with which the data are presented are arbitrary. In the present study, the instrument settings were generally optimized for maximal output signals of the various samples amounting to 3–5 Volt. The time-dependent fluorescence changes are plotted as relative fluorescence yield in arbitrary units, using the original voltage values for 540ex signals and appropriately rescaled values for 720ex signals (see section below on “Rescaling for comparison of 720ex and 540ex data”).

In the present study, the fluorescence responses induced by strong 540 nm multiple turnover pulses (MT), as measured with variously colored pulse-modulated ML, play a central role. The MT-induced rise of fluorescence yield consists of an initial “photochemical” phase from Fo to a first intermediate level I_1_ (O-I_1_ rise with rate proportional to quanta absorption by PSII) and two consecutive “thermal” phases, to a second intermediate level I_2_ and to a peak P (Delosme 1967, Schreiber 1986, Neubauer and Schreiber 1987, Schreiber and Neubauer 1987; for reviews, see Schreiber 2004, Lazar 2006, Stirbet and Govindjee 2012). As the actinic effect of the pulse-modulated ML is negligibly small, the physiological reactions induced in measurements with variously colored ML are equal and solely caused by the 540 nm MT. This is particularly true in view of the applied optical geometry (see Fig. 1), with which light gradients and their effects are minimized. Hence, any differences that are observed in the MT-induced responses using different colors of ML must be due to heterogeneous origins of the excited pulse-modulated fluorescence. In particular, such differences may be expected between the responses assessed with FR and visible ML, as the PSI/PSII excitation and resulting F(I)/F(II) ratios are substantially higher with FR than with visible light. In supplementary figure S1, PSI and PSII action spectra of the unicellular green alga *Scenedesmus obliquus* are shown (Schreiber and Vidaver 1974), from which a PSI/PSII excitation ratio spectrum was derived (figure S2), which is close to unity between 660 and 680 nm and thereafter increases by about a factor of ten. It may be assumed that the *Chlorella* used in the present study displays similar PSI/PSII excitation properties. The obtained results show, however, that the ratio of F(I)/F(II) derived from F > 765 measurements with 720ex and 540ex does *not* follow the expected PSI/PSII excitation ratios, which will be dealt with in the Discussion (see section on “Apparently “too small” F(I)/F(II) excitation ratio with 720ex”).

### Rescaling for comparison of the 720ex and 540ex data

The amplitudes of the original 720ex and 540ex signals are not directly comparable, as different ML intensities and different amplifier gains were used. For comparison of the changes of relative fluorescence yield, they had to be appropriately rescaled. For this purpose, a special routine was applied, the rationale of which is as follows:

It is generally accepted that on the one hand the O-I_1_ fluorescence rise constitutes a specific PS II response, Fv(II). This means that the F(II) changes with 720ex can be made equal to those with 540ex by multiplying all 720ex data points by an “equalization factor” such that the amplitude of O-I_1_ with 720ex becomes equal to the amplitude of O-I_1_ with 540ex (O-I_1_ equalization). Consequently, the thus rescaled O-I_1_-I_2_-P rise curves are termed to be “O-I_1_ equalized” or “O-I_1_ normalized.” In the context of the present study, the differences between the Fv with 720ex and the Fv with 540ex are of primary interest, as these are assumed to reflect Fv(I). The excess of Fv(I) measured with 720ex compared to 540ex is termed “extra Fv(I)” which is shown to consist of an “extra I_2_-P.” Hence, both terms will be used synonymously.

The rationale of O-I_1_ equalization and “extra Fv(I)” determination relies on the assumption that the signals obtained with 720ex and 540ex originate from the same cells in the same physiological states. Ideally, for this purpose both signals should be measured simultaneously. In reality, however, the two signals were measured alternatingly (i.e., 720ex alternating with 540ex with 5-min time intervals between measurements) under stationary physiologically stable conditions. In this context, it has to be remembered that *in vivo* during the dark between measurements reverse electron transport reactions take place, which influence the redox state of the intersystem PQ pool as well as of the ferredoxin-NADP pool at the PS I acceptor side. The O-I_1_ equalization factor was determined for groups of recordings that displayed stable stationary responses. This equalization factor showed small changes in the order of 10% during the course of a day.

In addition, for comparison of various O-I_1_ equalized responses, these could be normalized at the I_1_ level, such that, e.g., the amplitude of the “extra I_2_-P” is indicated as fraction of the O-I_1_ amplitude.

Systematic trivial differences between 720 and 540ex recordings may be expected when above a certain level of optical density the intensity gradients for the ML, AL, and MT start to disturb the measurements. In particular, if e.g., the mean PAR of the multiple turnover pulse of light that drives the polyphasic fluorescence rise would be lower with 720ex compared to 540ex, this would lead to apparent negative changes of Fv(I) during the course of the O-I_1_ rise. Due to the particular optical geometry developed for the present study (see block diagram of set-up in Fig. 1), even in the case of non-diluted deeply colored suspensions (of *Chlorella* as well as of *Synechococcus leopoliensis*) the data of the present study do not display much divergence between the O-I_1_ normalized 720ex and 540ex initial rise kinetics (see data below).

### Data variability and processing

The accuracy of the data presented in this study is limited by the variability in the recordings of the O-I_1_-I_2_-P kinetics. The actual measurements were fully automated (under the control of the dedicated PamWin-3 program) and highly accurate, with electronic noise of single recordings amounting to less than 2% of maximal fluorescence signals. For even higher accuracy a number of recordings were averaged. Somewhat larger variations may be caused by biological/physiological factors. This source of variability, however, did not affect the general observations and conclusions.

A defined redox state of the plastoquinone (PQ) pool was essential for high reproducibility of the O-I_1_-I_2_-P kinetics. It strongly depended on the “history” of preillumination, i.e., the time during the day–night cycle, time between consecutive measurements, etc. Use of weak FR (730 nm) background light proved useful to establish a defined PQ redox state before and between consecutive measurements (see section on “Photosynthetic organisms and sample preparation” below). Furthermore, in order to assure maximal comparability between 720 and 540ex, these responses were measured alternatingly with fixed 5-min intervals. The 720ex and 540ex responses were averaged separately by the PamWin-3 program. With each measurement running over 600 ms a total of 32,000 data points were saved by the program. The averaged data were exported to Excel, where the final plots were prepared.

### Photosynthetic organisms and sample preparation

All data except for those in Fig. 13 were measured with suspensions of the green unicellular alga *Chlorella vulgaris. Chlorell*a was cultured in natural day light (north window) at 20–40 µmol m^−2^ s^−1^ and ambient temperature (20–25 °C) in BG11 medium under ambient air. For the measurement in Fig. 13a, *Synechococcus leopoliensis* (former *Anacystis nidulans*) was grown photoautotrophically in BG11 medium under artificial light (warm white LED) at 30 °C. The batch cultures were shaken manually at least 4 times per day and frequently diluted so that the Chl content did not exceed 10 mg l^−1^. All experiments were carried out at room temperature (20–22 °C) with 1.3 ml aliquots of the non-diluted stock suspension at Chl concentrations of 8–10 mg l^−1^, as determined with a calibrated WATER-PAM chlorophyll fluorometer (Heinz Walz GmbH, Effeltrich, Germany). The suspensions within the 10 × 10 mm cuvette were continuously stirred with the help of a small magnetic “flea.”

For the measurements with moss, fern, and higher plant leaves presented in Fig. 13, the samples were fixed with double-sided tape on a 14 × 10 mm frame, which was placed at 45° in the optical center of the set-up displayed in Fig. 1, replacing the cuvette. Best results were obtained with the adaxial surfaces directed towards the 540 nm actinic source and the 540 nm ML, so that the abaxial surfaces were facing the FR-ML and the detector. The moss and fern were collected from a forest and the *Festuca arundinacea* (grass) from a vineyard near Würzburg-Heidingsfeld. Young light green leaves of *Ginkgo biloba* (Würzburg, Steinbachtal) and *Ficus benjamina* (potted indoor plant) were used.

## Results and interpretation

### Identification of Fv(I) by comparative recordings of light-induced changes measured with 720 nm and 540 nm pulse-modulated excitation

Using the experimental set-up depicted in Fig. 1, the polyphasic fluorescence rise kinetics induced upon onset of strong actinic illumination was measured in dark-adapted *Chlorella* with 720 nm (720ex) and 540 nm (540ex) pulse-modulated excitation (ML). The 720ex and 540ex measurements were carried out in close to equal physiological states: Repetitive recordings of 720ex and 540ex were alternated (each involving a 600 ms MT pulse of strong 540 nm light) with 5-min dark intervals in between, until stable stationary responses were obtained. Then 4 recordings each were averaged. Figure 4 shows a screenshot of the original averaged responses with logarithmic time scale. The ML intensities and amplifier gains of the two signals were adjusted such that the amplitudes of the initial rise of fluorescence yields from the Fo to the I_1_ level (“photochemical phase,” O-I_1_) were about equal. As there is general agreement that O-I_1_ reflects the closure of PS II reaction centers upon reduction of the primary stable acceptor Q_A_, O-I_1_ may be considered a specific indicator of Fv(II). If the same would also be true for the following “thermal phase” of the fluorescence rise, this should be equal with 720ex (red curve) and 540ex (blue curve) as well. All changes of fluorescence originating from the same photosystem should be proportional to each other. While at first sight this indeed appears to be the case, a closer look at the data in Fig. 4 reveals, however, that the fluorescence rise from I_1_ to P (i.e., the “thermal phase”) is somewhat larger with 720ex compared to 540ex: In the time courses of the MT-induced responses, the difference between the two signals remains constant at 675 mV up to 2 ms (end of the photochemical phase) and then increases to 755 mV during the thermal rise to the P level. As the PSI/PSII excitation ratio undoubtedly is higher with 720ex compared with 540ex, this means that part of the fluorescence rise to the P level must originate from PS I and, hence, reflects Fv(I). This finding is in line with the previous theoretical considerations of Lazar (2013) and experimental evidence obtained from comparative measurements of polyphasic rise kinetics of F > 700 and F < 710 (Schreiber and Klughammer 2021).

A surprising new finding is that even with 720ex, which commonly is assumed to give very low excitation of PSII, a large amplitude O-I_1_ rise is observed. Furthermore, the transient peak at 150–200 ms, which in Schreiber and Klughammer (2021) was suggested to be due to Fv(I) is smaller than expected. In view of previously measured PSI and PSII action spectra (Schreiber and Vidaver 1974; Laisk et al. 2014; see also supplementary figures S1, S2) intuitively it was expected that the ratio of Fv(I) to Fv(II) would increase dramatically using excitation wavelengths > 700 nm. Not only are wavelengths > 700 nm more strongly absorbed by PS I, but in addition it has to be considered that the observed increase of fluorescence yield upon closure of PS II means that the excitons generated by 720ex can cause charge separation in PS II. Otherwise they could not respond to the closure of PSII. Hence, while the data in Fig. 4 in principle agree with the expectation that Fv(I)/Fv(II) should increase when fluorescence excitation is moved from the visible to the FR spectral range, this increase appears surprisingly small. Possible explanations will be considered in the Discussion.

### Assessment of Fo(720ex) and the ratio of Fo(I)/Fo(II) with 720ex

An increase of the PS I/PS II excitation ratio with 720ex with respect to 540ex should not only be reflected in the Fv(I) and Fv(II) components, but to the same extent also in the amplitudes of Fo(720ex) and Fo(540ex), both of which are composed of Fo(I) and Fo(II). When after O-I_1_ normalization, all responses originating from PSII are equalized, also Fo(II)720ex and Fo(II) 540ex should be equal. On the other hand, with increased PS I/PS II excitation ratio, the Fo(I)720ex/ Fo(I)540ex ratio should be correspondingly larger. The question is whether, after equalizing Fo(II)720ex and Fo(II)540ex, this ratio is as high as expected from the known PSI/PSII ratio. For a quantitative treatment of this aspect, reliable assessment of the overall Fo values is required which in the case of Fo(720ex) is complicated by a relatively large background signal that is mainly caused by scattered and reflected 720 nm ML. It was attempted to correct for this background signal by estimating its contribution from the signal observed in the presence of strongly scattering BaSO_4_ (see Materials and Methods, supplementary figures S3–S5). The broken red line in Fig. 4 shows the corrected baseline for 720ex, based on which Fo (720ex) can be assessed.

**Fig. 5:**
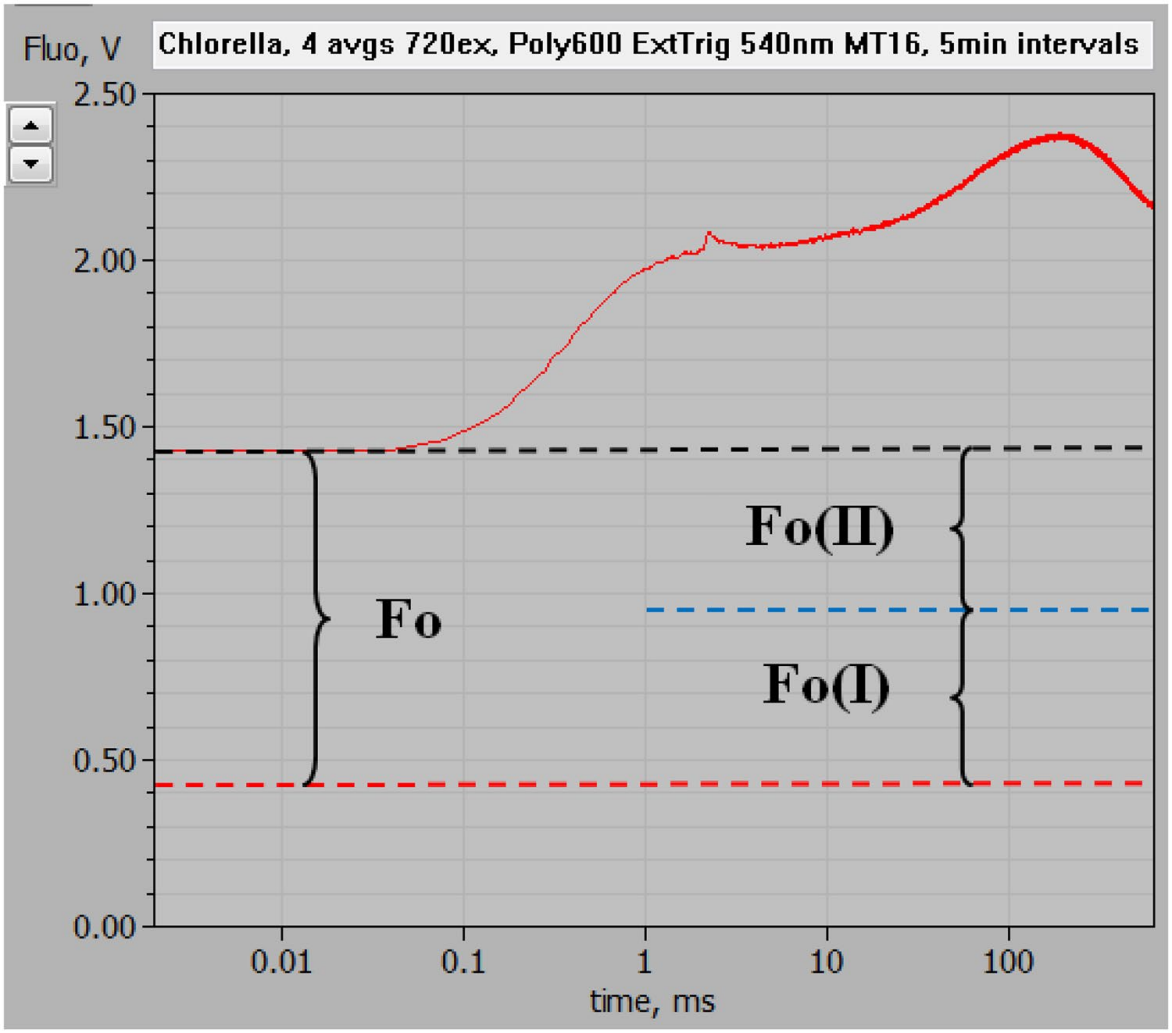
Polyphasic fluorescence rise upon onset of strong actinic illumination measured with dark-adapted *Chlorella *using 720 nm pulse-modulated excitation, as also shown in Fig. 4, with deconvolution of Fo into the contributions of Fo(I) and Fo(II). The corrected baseline defined by the estimated background signal (see supplementary figures S3–S5) is indicated by the broken red line. For information on the rationale of deconvolution, see text and supplementary figures S6–S7

The Fo(I) contribution to the overall Fo measured with 720ex can be derived, based on a plausible estimate of the Fo(I)/Fo with 540ex. Previous work from several laboratories has suggested contributions in the order of 30–40% of Fo(I) at wavelengths > 700 nm upon excitation with visible light in C3 organisms (Genty et al. 1990; Pfündel 1998; Peterson et al. 2014). When it is assumed that Fo (540ex) in the experiment of Fig. 4 consists of 35% Fo(I) and 65% Fo(II), it follows that the Fo (720ex) is composed of 52% Fo(I) and 48% Fo(II), as outlined in supplementary figures S6–S7 and depicted in Fig. 5. While with 540ex Fo(I)/Fo(II) = 35/65 = 0.539, it amounts to 52/48 = 1.083 with 720ex. Hence, the Fo(I)/Fo(II) ratio with 720ex happens to be almost exactly 2 × higher than with 540ex. Consequently, also the ratio of Fv(I)/Fv(II) should be 2 × higher with 720ex compared with 540ex. Based on this assumption, which should be generally valid for dark-adapted *Chlorella* under the given culture conditions, the Fv(I) and Fv(II) components of the polyphasic rise kinetics can be derived, as outlined in the following section on “Deconvolution of Fv(I) and Fv(II).”

### Deconvolution of Fv(I) and Fv(II)

**Fig. 6:**
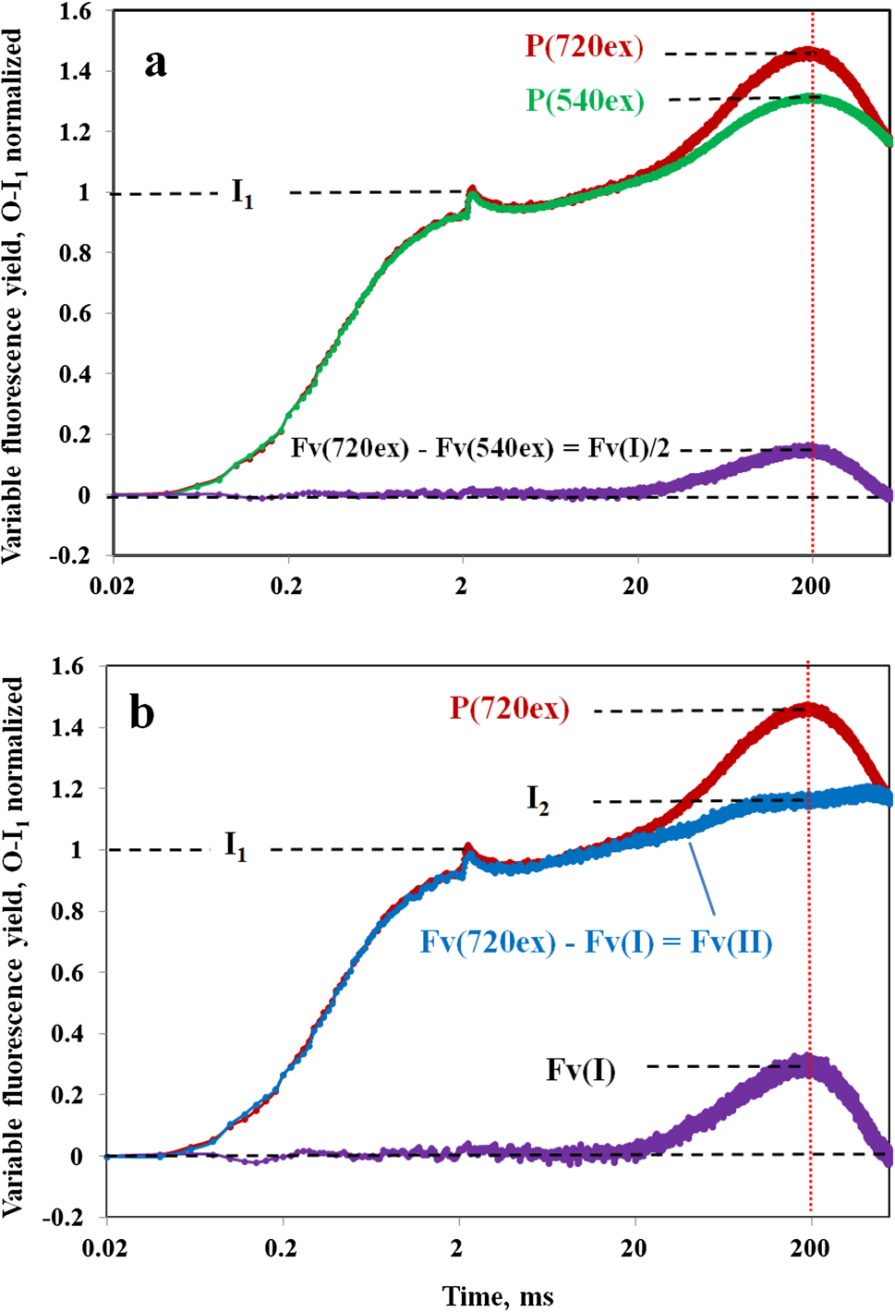
Deconvolution of the Fv(I) and Fv(II) components of the overall Fv(720ex) polyphasic rise kinetics in *Chlorella* after dark-adaptation (PQ pool partially reduced). **a** Normalization of Fv(720ex) (red trace) and Fv(540ex) (green trace) at the I_1_ level, defined at 2 ms (end of the photochemical phase) by a saturating single turnover flash. Derived from original data presented in Fig. 4. With all F(II) components being equal after O-I_1_ normalization, the difference curve (violet trace) corresponds to Fv(I)/2 contained in Fv(720ex) (see text). **b** Deconvolution of Fv(II)(blue trace) from the difference between Fv(720ex) and the Fv(I) derived from Fv(I)/2 in panel a. Definition of the I_2_ level so that at the Fv(I) peak (200 ms, vertical red dotted line) I_2_ = P(720ex)−Fv(I)

Figure 6A shows the Fv part of the 720ex and 540ex responses displayed in Fig. 4 after O-I_1_ normalization, i.e., after equalization of Fv(II). In this form of presentation, the differences between the 720ex (red) and 540ex (green) curves can be better evaluated. The difference curve (violet) reflects the kinetics of Fv(I), with the amplitude amounting to the “extra Fv(I)” that is contained in the 720ex compared to the 540ex response, Fv(720ex) – Fv(540ex). As outlined in the preceding section on “Assessment of Fo(720ex) and the ratio of Fo(I)/Fo(II) with 720ex,” it may be expected that after O-I_1_ equalization Fv(I)720ex/Fv(I)540ex = 2 and, hence, that the “extra Fv(I)” corresponds to half of the total Fv(I) contained in the 720ex response and that it equals the Fv(I) contained in the 540ex response. In *Chlorella* under the given conditions the peaks of the 720ex and 540ex responses coincide with the peak of Fv(I) almost exactly at 200 ms.

In Fig. 6b the Fv(I) and Fv(II) responses are displayed together with the overall 720ex response, from which they were deconvoluted. The kinetics of Fv(II) were derived by subtracting Fv(I) from the overall response. Analogously, also the 540ex response can be deconvoluted, as shown in supplementary figure S10.

The data in Fig. 6 show that in *Chlorella* Fv(I) starts rising at about 20 ms after onset of strong illumination, where under the given experimental conditions the increase of Fv(II) is not yet completed. Up to this time the 720ex, 540ex, and Fv(II) responses are close to equal. The Fv(I) response is transient, reaching a peak at about 200 ms and declining to zero within the following 400 ms. In the 20–200 ms time range, the rise of Fv(720ex) is much more pronounced than that of Fv(II).

The measurements of Figs. 4–6 were after dark-adaptation that traditionally has been applied for the study of dark–light induction transients, as also in the pioneering study of Delosme (1967), in which René Delosme described the separation of a photochemical rise phase from Fo to an intermediate level I (O-I transient) and a single thermal phase with fluorescence rising from I to a peak, P, (I-P transient) using a *Chlorella* suspension (see Fig. 4 in Delosme 1967). Later measurements with leaves and isolated chloroplasts revealed the existence of two intermediate levels, I_1_ and I_2_, splitting the “thermal phase” into two sub-phases, I_1_-I_2_ and I_2_-P, that display distinctly differing properties (Schreiber 1986; Neubauer and Schreiber 1987; Schreiber and Neubauer 1987). As we know now, in suspensions of algae and cyanobacteria the intersystem plastoquinone (PQ) pool becomes partially reduced after dark-adaptation, leading to an increase of I_1_ towards the level of I_2_, thus largely eliminating the I_1_-I_2_ phase. This is the reason why identification of an I_2_ step or inflection is problematic in the case of Figs. 4–6. While the I_1_ level can be determined accurately with the help of a saturating single turnover flash, definition of the I_2_ level is difficult, particularly when the PQ pool is pre-reduced, so that the amplitude of the thermal phase preceding the Fv(I) rise is rather small and no clear-cut step or inflection separating the I_1_-I_2_ and I_2_-P phases is apparent.

The polyphasic fluorescence rise essentially describes the filling of three consecutive electron acceptor pools, namely primary PSII acceptors Q_A_ and Q_B_ (O-I_1_), intersystem PQ pool (I_1_-I_2_), and PSI acceptor pool (I_2_-P). At high light intensity, full reduction of Q_A_ and Q_B_ can be readily achieved before any significant PQ reduction occurs. On the other hand, both the reduction of the PQ pool and of the PSI acceptor pool are limited by thermal reactions. As PQ reoxidation at the Cyt b/f complex limits the overall rate, filling of the PQ pool precedes that of the PSI acceptor pool. However, as suggested by the data in Fig. 6b, the former may not be completed, before the latter sets in. Based on the quantitative information obtained above on the composition of Fo with 720ex and 540ex (see preceding section), a straight forward definition of the I_2_ level for the given data is possible by assuming that I_2_ = P(720ex)−Fv(I). As shown in Fig. 6b, the thus defined I_2_ level corresponds to the transient plateau of Fv(II) in the time range, where Fv(I) shows a peak.

**Fig. 7:**
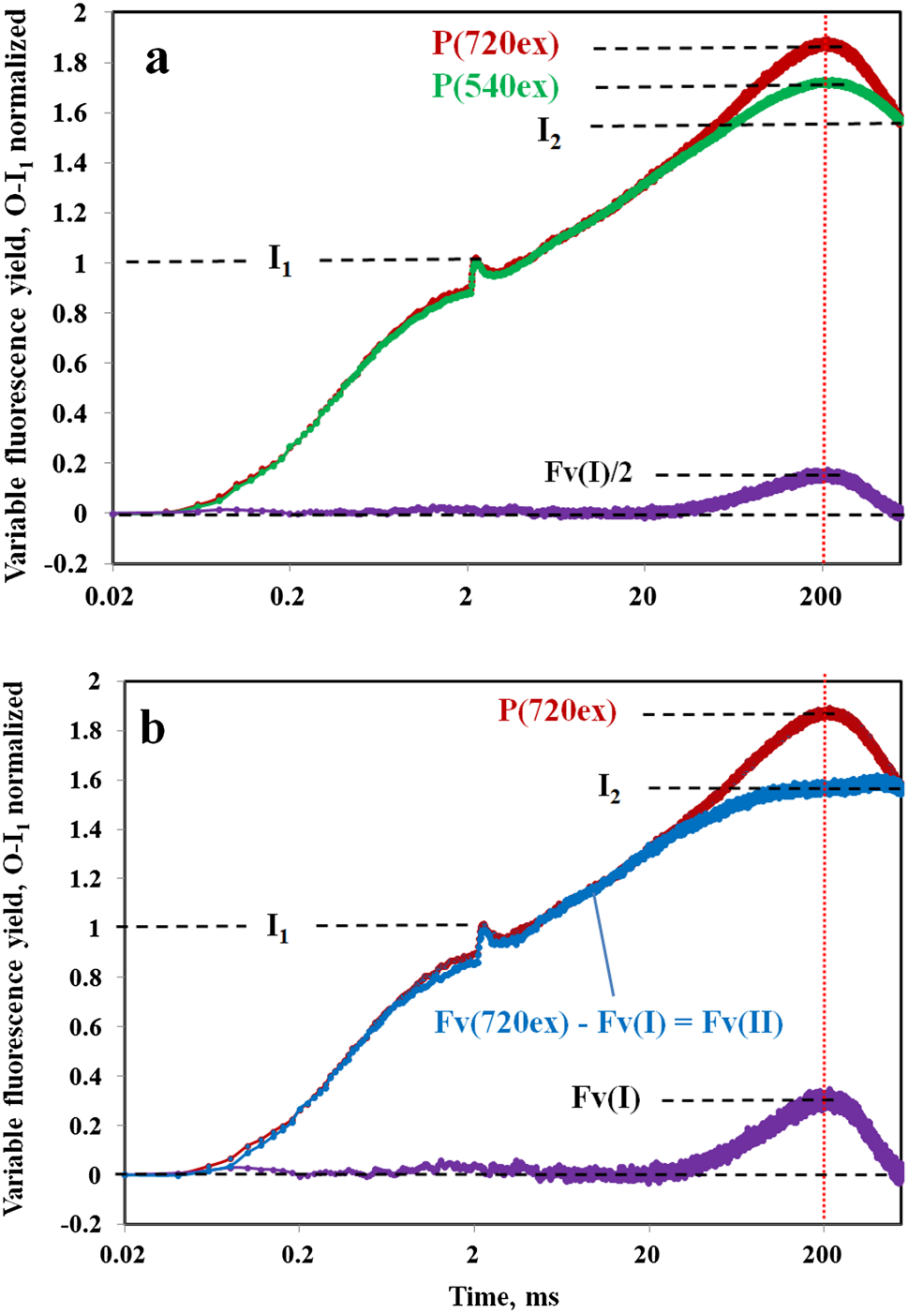
Deconvolution of the Fv(I) and Fv(II) components of the overall Fv(720ex) kinetics in the presence of weak FR background light (PQ pool pre-oxidized). **a** Normalization of Fv(720ex) (red trace) and Fv(540ex) (green trace) at the I_1_ level, defined at 2 ms (end of the photochemical phase) by a saturating single turnover flash. Same sample as in measurements of Fig. 6, after 15-min adaptation to 1 µmol m^−2^ s^−1^ 730 nm quanta. With all F(II) components being equal after O-I_1_ normalization, the difference curve (violet trace) corresponds to Fv(I)/2 contained in Fv(720ex) (see text). **b** Deconvolution of Fv(II)(blue trace) from the difference between Fv(720ex) and the Fv(I) derived in panel a. Definition of the I_2_ level so that at the Fv(I) peak (200 ms, vertical red dotted line) I_2_ = P(720ex)−Fv(I)

In the dark-adapted state, very low intensity of FR background light suffices to oxidize the PQ pool in *Chlorella*. As shown in Fig. 7, this leads to a substantial decrease of the I_1_ level and a corresponding increase of the thermal phase preceding the Fv(I) rise, with indication of an I_2_ inflection. Based on this inflection, however, an *accurate* determination of the I_2_ level and, hence, of the amplitude of the I_2_-P phase still would be difficult. Instead, analogously to Fig. 6, it is assumed that the “extra Fv(I)” derived from the difference of the O-I_1_ normalized Fv(720ex) and Fv(540ex) responses equals half of the Fv(I) contained in the 720ex curve and that I_2_ = P(720ex)−Fv(I). Hence, in Fig. 7 the I_2_ level is indicated at Fv(I)/2 distance from P(540ex) (panel a) and at Fv(I) distance from P(720ex) (panels a and b). The thus defined I_2_ level coincides with the equal amplitudes of F(720ex), F(540ex), and Fv(II) close to the end of the recording, where the Fv(I) transient has declined to zero. With the above definition of the I_2_ level, the presented data are in line with the notion that in *Chlorella* the whole fluorescence increase from I_2_ to P is due to Fv(I), as originally suggested by Schreiber and Klughammer (2021).

It is important to note that the deconvolution of Fv(I) and Fv(II) presented in Figs. 6 and 7 depends on information on the F(I)/F(II) excitation ratios with 720ex and 540ex, specific for a particular sample under the given conditions. While such information could be obtained for *Chlorella* (see preceding section and supplementary figures S3–S5) it is not readily available for other photosynthetic organisms. It remains to be investigated to what extent the value of Fo(I)720ex/Fo(I)540ex = 2 may vary between different photosynthetic organisms.

When information on the F(I)/F(II) excitation ratios is *not* available, assessment of the Fv(I) amplitude has to rely solely on the “extra Fv(I),” i.e., the difference between the O-I_1_ equalized Fv(720ex) and Fv(540ex) responses. In this case, an *empirical* determination is possible: The factor by which the “extra Fv(I)” has to be multiplied to match the 720ex response in the P region of the kinetics can be readily determined by trial and error. In practical applications, once it is accepted that I_2_-P is due to Fv(I), this is the most simple approach.

It should be kept in mind that the value of Fo(I)720ex/Fo(I)540ex = 2 for dark-adapted *Chlorella* was determined under the assumption that Fo(540ex) consists of 0.35 Fo(I) and 0.65 Fo(II), which corresponds to an F(I)/F(II) excitation ratio of 0.538 with 540ex. In this context, it is of interest to know, how much the Fo(I) excitation ratio will diverge from 2, when values of Fo(I)540ex/Fo(540ex) are assumed that differ from 0.35 and how much this will effect the deconvoluted Fv(I) and Fv(II) responses. This question is answered in supplementary figures S8 and S9, where it is shown that from Fo(I)540ex/Fo(540ex) = 0.2 to 0.45 the value of Fo(I)720ex/Fo(I)540ex varies from 2.76 to 1.78. While a decrease of the latter from 2.00 to 1.78 just leads to a minor decrease of Fv(I) and a correspondingly small increase of Fv(II), an increase to 2.76 results in 30% stimulated Fv(I) and a “trough” in the Fv(II) kinetics, which is followed by an additional rise phase. Such Fv(II) kinetics seem unlikely and, therefore, there appears to be little room for Fo(I)720ex/Fo(I)540ex values much higher than 2 in *Chlorella*.

In practice, quantification of the absolute value of Fv(I) normally is of less interest than information on relative changes of Fv(I). Such information is given directly by the “extra Fv(I)” that is obtained without any bias on the F(I)/F(II) excitation ratio, by simply subtracting Fv(540ex) from Fv(720ex) after O-I_1_ equalization. An important aspect in practical applications is the stability of the physiological state that determines the F(I)/F(II) excitation ratio and the reactions at the PSI acceptor side *in vivo*. Fv(I) may be expected to be influenced by numerous parameters, the stability of which has to be ascertained. This aspect is of particular importance, as with the present measuring system the 720ex and 540ex responses cannot be measured simultaneously. For obtaining reproducible Fv(I) responses two methods have proven practicable:

1. Preillumination of the sample at moderate light intensity for 15–90 min, followed by application of extremely weak far-red background light (setting FR1 with PamWin-3 user software); start of alternating measurements with 720ex and 540ex about 15 min after end of preillumination, with 5-min intervals between consecutive measurements.
2. As described under (1), but without FR application. In this case, a slow constant flow of electrons from stroma reductants into the PQ pool maintains an almost constant high level of PQ reduction over 1–2 h, linked with a stable state 2 of energy distribution between the two photosystems.

The method (2) is applied in the following section to quantify the dependence of the “extra Fv(I)” on the wavelength of fluorescence excitation in the “red drop” spectral range between 680 and 740 nm.

### Development of “extra Fv(I)” at excitation wavelengths above 680 nm

**Fig. 8:**
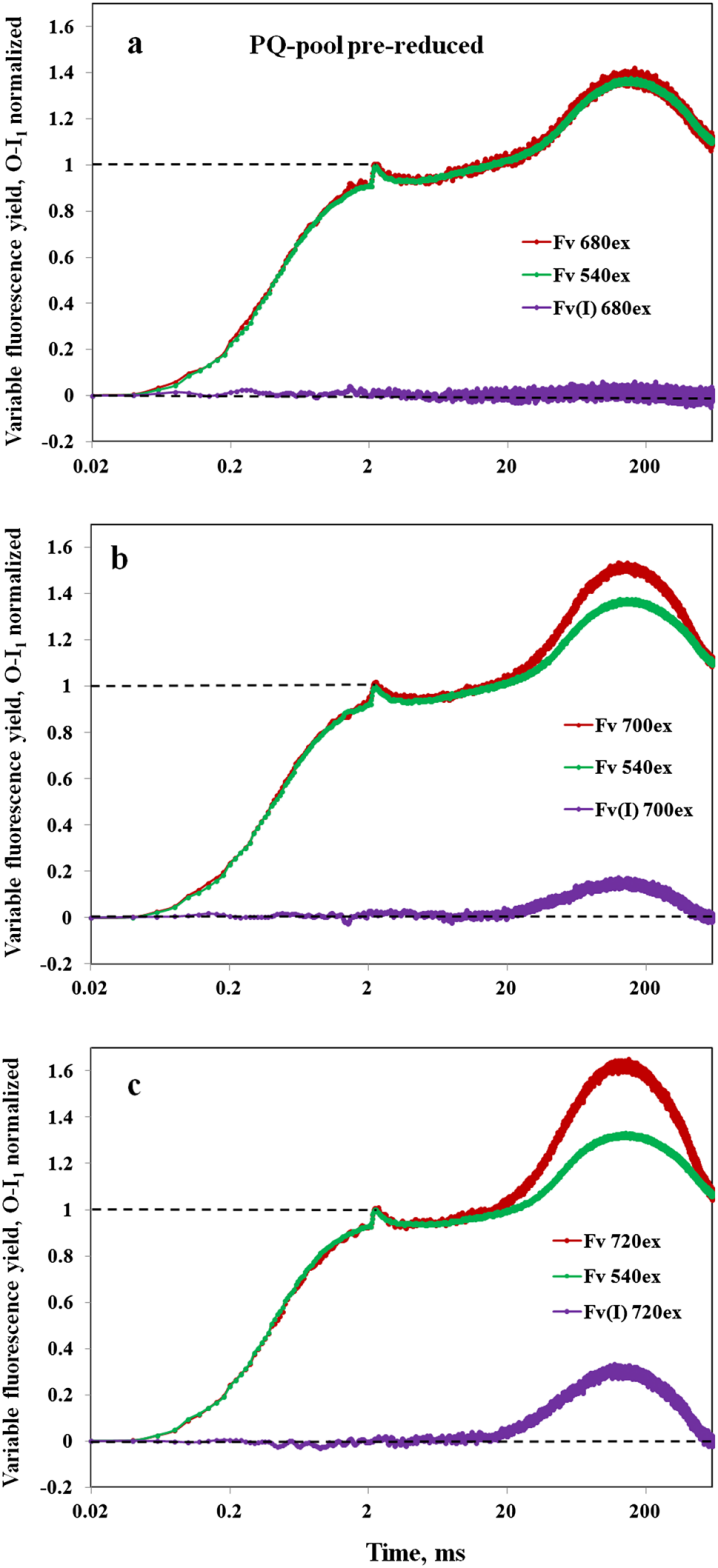
Polyphasic rise kinetics of *Chlorella* measured alternatingly with 540ex and 680ex (panel a) or 700ex (panel b) or 720ex (panel c). 5-min dark intervals between consecutive measurements. Due to a preceding 80-min continuous illumination at 715 µmol 540 nm quanta m^−2^ s^−^1, which was followed by 15-min darkness, the PQ pool was pre-reduced and the sample in state 2. The 540ex curves are the averages of one curve measured before and one curve measured after each respective 680ex, 700ex, or 720ex curve. Subtraction of the respective 540ex curves (green) from the O-I_1_ normalized 680ex, 700ex, and 720ex curves (dark red) yielded the kinetics of “extra Fv(I)” (violet). Polyphasic rise kinetics measured with 600 ms Multiple Turnover pulses (MT) of 540 nm light at 8000 µmol m^−2^ s^−1^

In Fig. 8 the O-I_1_ normalized responses with 680ex, 700ex, and 720ex are compared with the corresponding 540ex responses, measured under close to equal conditions. The sample had been preilluminated for 80 min with 540 nm AL at 715 µmol quanta m^−2^ s^−1^, followed by 15-min darkness, during which the PQ pool had become largely reduced and a stable state 2 was established. The green 540ex curves are the averages of the two 540ex responses measured 5 min before and 5 min after the responses with the other wavelengths (dark red), 680ex in panel a, 700ex in panel b, and 720ex in panel c. The “extra Fv(I)” curves (violet) correspond to the difference between the dark red and green curves, e.g., 720ex minus 540ex in panel c.

As apparent from Fig. 8a, there is hardly any difference between the O-I_1_ normalized 540ex and 680ex responses. The obvious lack of “extra Fv(I)” suggests that under the given conditions the F(I)/F(II) excitation ratios with 540 nm and 680 nm are close to equal in *Chlorella*. Distinctly enhanced “extra Fv(I)” amplitudes were observed with 700ex (panel b) and 720ex (panel c).

**Fig. 9:**
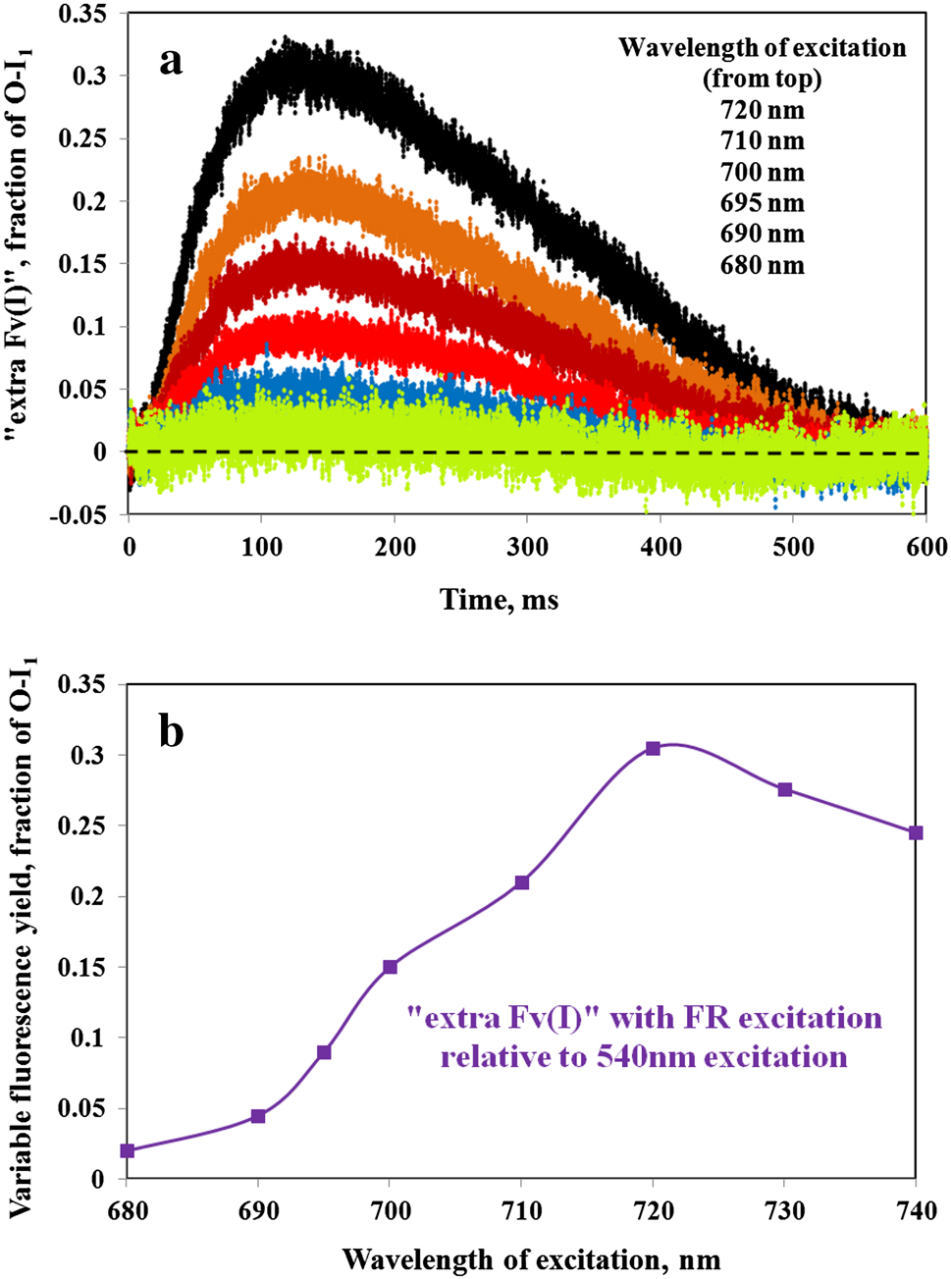
Information on the “extra Fv(I)” in *Chlorella* derived from comparative measurements of polyphasic rise kinetics using 540ex and a variety of excitation wavelengths in the red-FR spectral range. **a** Kinetics of “extra Fv(I).” **b** Amplitude of the “extra Fv(I)” as a function of the excitation wavelength in the red-FR range. The “extra Fv(I)” is scaled as fraction of the O-I_1_ amplitude

Analogous measurements were carried out with a variety of excitation wavelengths in the 680 to 740 nm spectral range and the “extra Fv(I)” amplitudes were derived from the respective O-I_1_ normalized curves. This is the “red drop” wavelength range, where the drop of PS I absorption is shifted by about 10 nm relative to the drop of PS II absorption (see supplementary figures S1, S2). The outcome of these measurements is presented in Fig. 9. Figure 9a shows the “extra Fv(I)” kinetics for 720ex, 710ex, 700ex, 695ex, 690ex, and 680ex, with “extra Fv(I)” scaled as fraction of O-I_1_. For clarity of presentation, the “extra Fv(I)” kinetics of 730ex and 740ex were omitted from this figure, as they partially overlap with the 720ex kinetics. Figure 9b shows a plot of the “extra Fv(I)” amplitude as a function of excitation wavelength (always derived from the difference of the FRex and 540ex responses after O-I_1_ normalization).

The kinetics of the “extra Fv(I)” observed with the different excitation wavelengths (panel a) are very similar, just differing in the maximal amplitudes of the “extra Fv(I).” This is not unexpected, as the driving force for the changes of Fv(I) in all cases is the same, namely the 600 ms 540 nm multiple turnover pulse (MT) (saturating with respect to inducing a maximal increase of fluorescence yield, from the Fo to the P level). Under the given conditions (preilluminated sample, PQ pool pre-reduced, state 2, 21 °C) Fv(I) already reaches a peak at about 130 ms following the onset of the MT and then declines again with a half-time of about 300 ms. Hence, as emphasized before (Schreiber and Klughammer 2021), Fv(I) is a transient phenomenon, observable just in a short window of time during a pulse of saturating light.

As depicted in Fig. 9b, the increase of the “extra Fv(I)” between 680 and 720 nm consists of two steps, which are reminiscent of the two steps in the increase of the PS I/ PS II action spectrum derived from the *Scenedesmus* data of Schreiber and Vidaver (1974) (see supplementary figures S1, S2). While the first step appears to be primarily due to an increase of PS I excitation, the second step falls into the range where both PS I and PS II excitation drop, but PS II more than PS I. Interestingly, a peak of the F(I)/F(II) excitation ratio seems to be reached at 720 nm. These data confirm that Fv(I) develops in parallel with the well-known increase of the PSI/PSII excitation ratio.

### Dependence of Fv(I) on actinic intensity

**Fig. 10:**
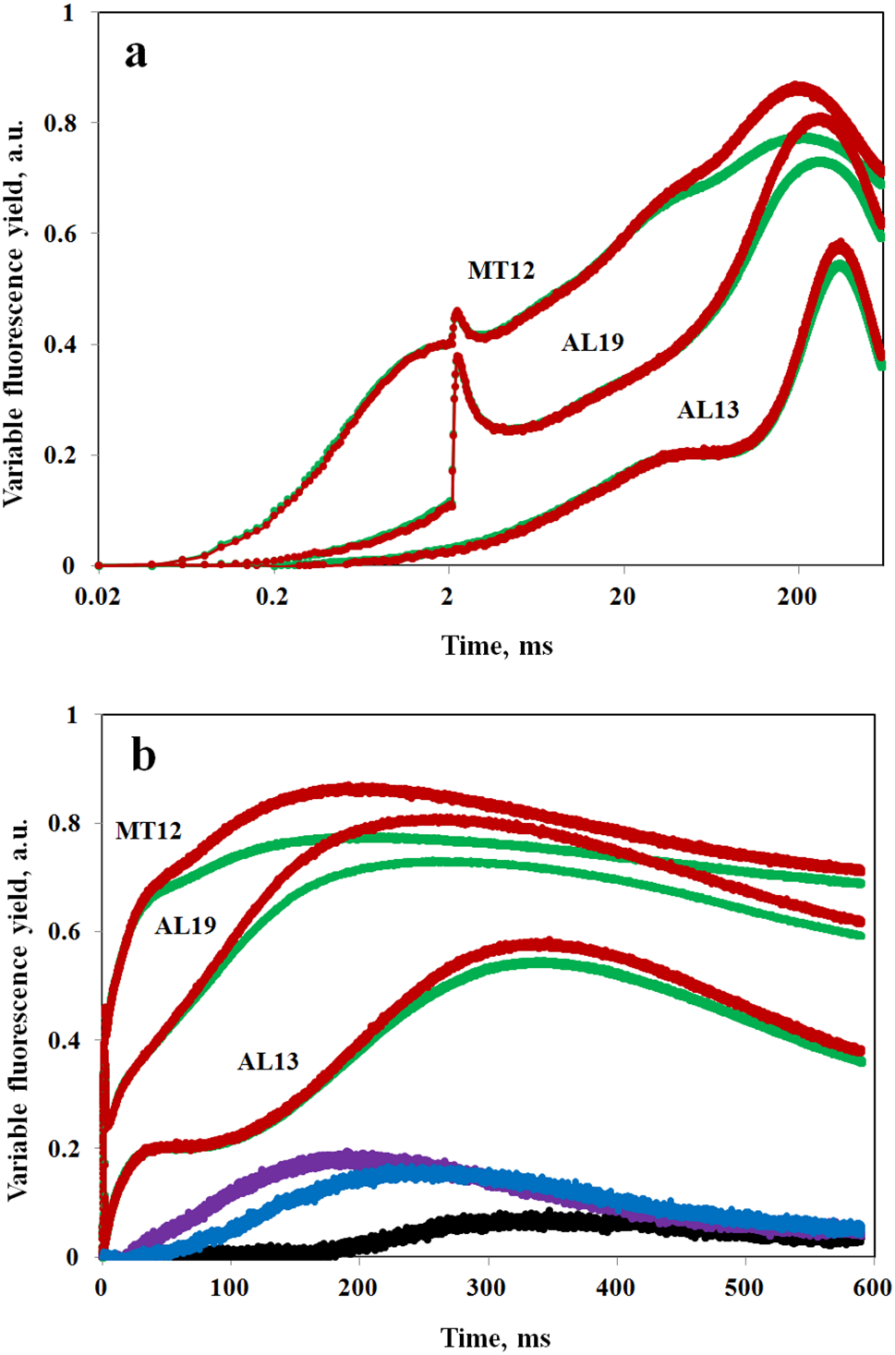
Dark–light fluorescence induction kinetics upon onset of 540 nm actinic illumination at 3 different intensities measured with 720ex (red traces) and 540ex (green traces). *Chlorella* in the presence of weak FR background light (PQ pool pre-oxidized), as in the experiment of Fig. 7. Actinic intensities in µmol m^−2^ s^−1^ 540 nm quanta: AL13, 445; AL19, 1665, and MT12, 6150. Averages of 10 recordings each measured alternatingly using 720ex and 540ex with 3-min dark intervals in between. AL19 and MT12 recordings with application of saturating single turnover flash at 2 ms. Normalization of 720ex and 540ex responses at the end of the initial photochemical phase: with AL19 and MT12 at I_1_, with AL13 at I level. **a** Logarithmic time scale. **b** Linear time scale. The Fv(I) responses for AL13 (black), AL19 (blue), and MT12 (violet) are displayed at the bottom, as calculated from [ Fv(720ex)−Fv(540ex)]*2

In the measurements of Figs. 4–9, the dark–light induction kinetics were driven by 8000 µmol m^−2^ s^−1^ 540 nm quanta. Such high-quantum flux density has proven necessary for optimal differentiation between the “photochemical” O-I_1_ phase and the two “thermal” phases (I_1_-I_2_ and I_2_-P) in the polyphasic rise kinetics (see e.g., Fig. 4 in Schreiber 1986). The question arises: How much Fv(I) may be hidden in dark–light induction curves that are measured at *moderate* actinic intensities, when no I_2_-P phase can be distinguished? An answer to this question is given in Fig. 10. Appreciable Fv(I) already is observed at AL19 (1665 µmol m^−2^ s^−1^ 540 nm quanta), where no I_2_-P inflection is apparent. As to the applied quantum flux density, it has to be considered that the wavelength-dependent functional cross-section of PSII is about six times smaller with 540ex compared to 440ex (Schreiber et al. 2012). At AL13 (445 µmol m^−2^ s^−1^ 540 nm quanta) a “classical” Kautsky effect with O-I-P-S transients is measured (Kautsky and Franck 1948; Munday and Govindjee 1969; Franck et al. 1969; Govindjee 1995). It is apparent that even at this moderate intensity, a small but significant part of the I-P-S transient is due to Fv(I).

The data presented in Fig. 10 may help to understand the relationship between the “classical” I-P-S transient measured at moderate actinic intensity and the I_1_-I_2_-P-S transients of the polyphasic rise kinetics in saturating light. In this context, the following points shall be emphasized:

1. The I-P-S transient at AL13 is dominated by Fv(II) that is indirectly controlled by the PQ redox state. At this moderate intensity, it takes about 100 ms until fluorescence yield starts rising beyond the I level, i.e., until the rate of Q_A_ reoxidation by Q_B_ begins to decline. Fv(I) develops distinctly later at about 200 ms.
2. While with increasing intensity, the filling of both the PSII and the PSI acceptor pools is speeded up, the former is somewhat faster than the latter. This is important for the feature of the I_2_ inflection, which indicates saturation of Fv(II), kinetically preceding the main increase of Fv(I).
3. The relative rates with which the PSII and PSI acceptor pools are filled upon sudden application of saturating light may be expected to depend on the species and a number of environmental/physiological parameters. The extent of the I_2_ inflection or step should be favored by a high ratio of PSI/PSII acceptor pool sizes and a low ratio of PSI/PSII excitation. Depending on these parameters, there can be more or less separation between the complete closure of PSII and the beginning closure of PSI. In this context, also cyclic PSI may play a role, details of which have to be elucidated by further research.

### Suppression of I_2_-P and Fv(I) by DCMU

**Fig. 11:**
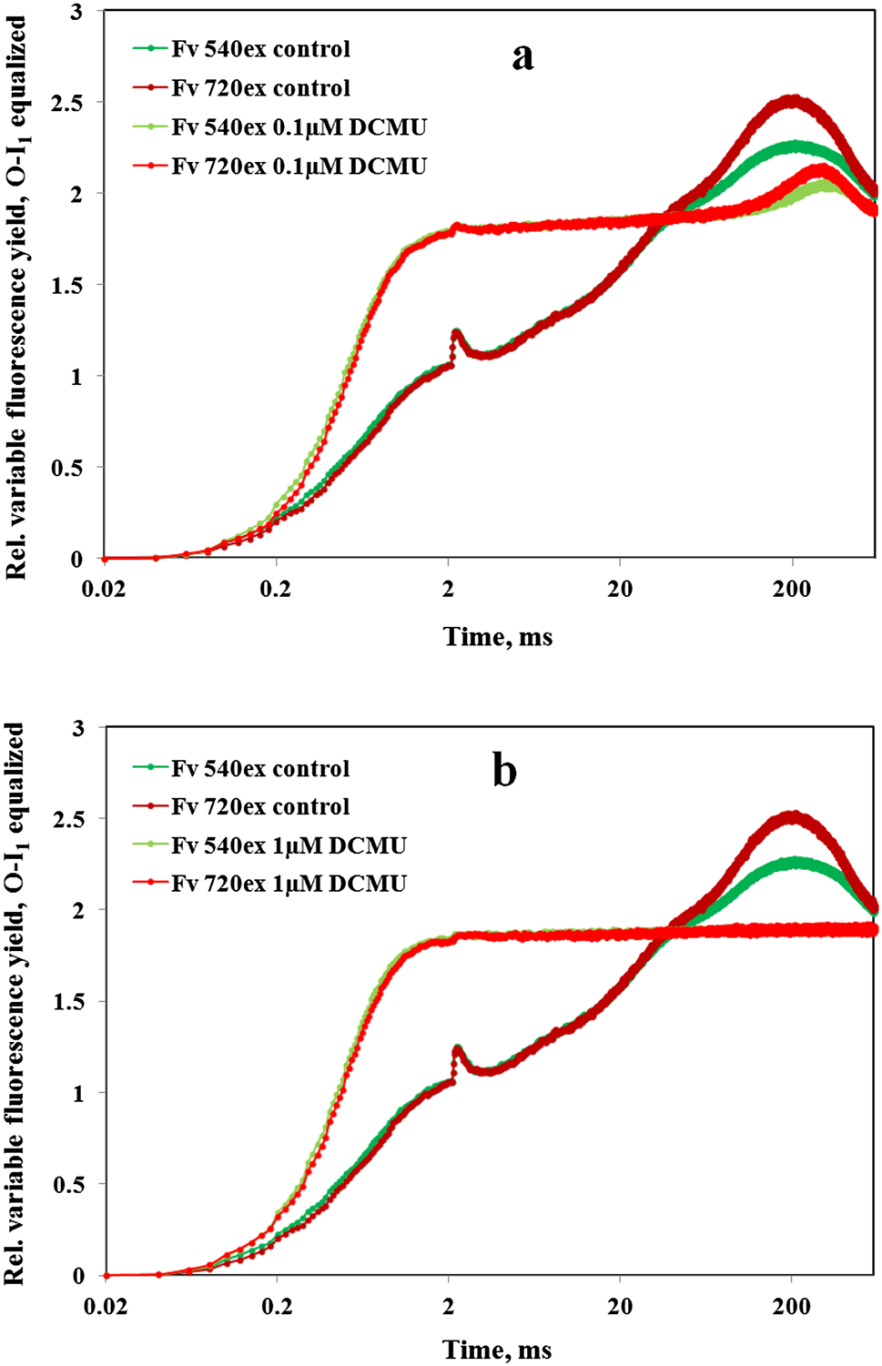
Effects of 0.1 µM DCMU (panel a) and 1 µM DCMU (panel b) on the polyphasic rise kinetics measured with 720ex and 540ex. Application of weak 730 nm background light (1 µmol m^−2^ s^−1^) to keep PQ pool oxidized. Actinic illumination with 600 ms Multiple Turnover pulse (MT) of 540 nm light at 5200 µmol m^−2^ s^−1^. Using one and the same sample first the control curves were measured, then the 0.1 µM DCMU curves after 60-min incubation and finally the 1 µM DCMU curves after 60-min incubation. Two curves each with 720ex were averaged which were measured 5 min before and 5 min after recording of the 540ex curve. Control, 0.1 µM, and 1 µM DCMU curves were separately O-I_1_ equalized

**Fig. 12:**
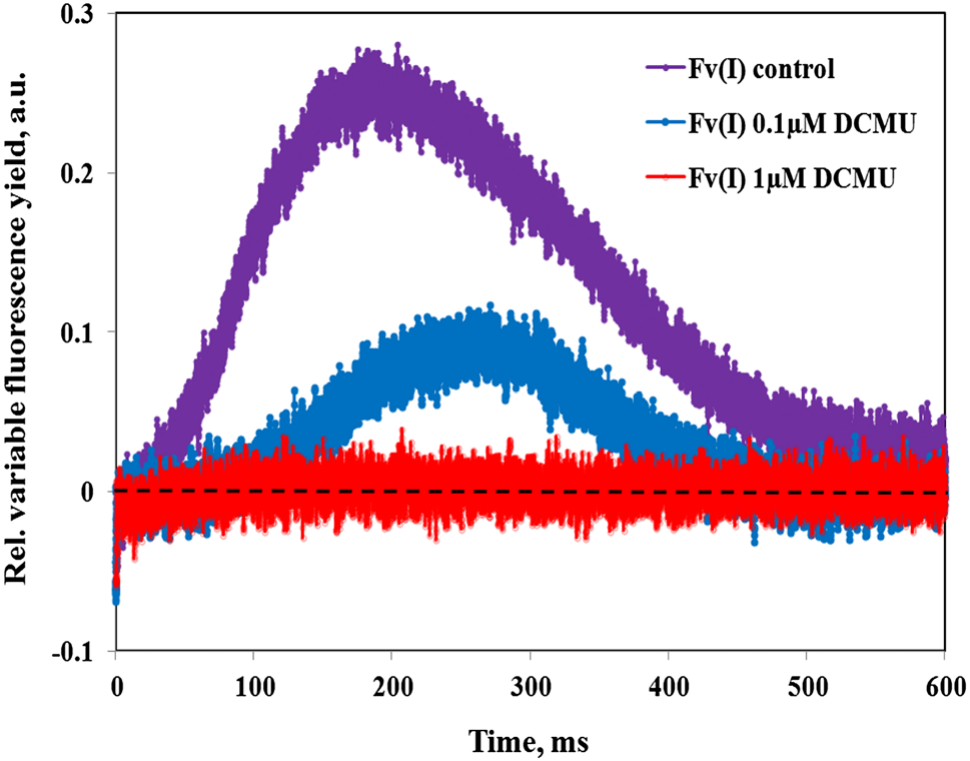
Kinetics of “extra Fv(I)” derived from the O-I_1_ equalized polyphasic rise kinetics measured with 720ex and 540ex in Fig. 11 by subtracting the 540ex curves from the 720ex curves

Toth et al. (2005) reported “identical Fm values with and without DCMU” based on careful measurements with intact leaves, in which the PSII inhibitor was applied in total darkness and allowed to diffuse slowly into the leaf tissue. If Fm were equal ± DCMU also in *Chlorella*, this would seriously question the notion of I_2_-P reflecting Fv(I). In Figs. 11 and 12, the effects of 0.1 µM and 1 µM DCMU on the polyphasic rise kinetics measured with 720ex and 540ex are shown. The same weak 730 nm background light (FR1) as in the experiments of Figs. 7 and 10 was applied, to keep the PQ pool as well as the secondary PS II acceptor Q_B_ oxidized before and during incubation with DCMU. In this way, an increase of Fo upon displacement of Q_B_ from its binding site by DCMU (Velthuys 1981) was minimized.

From the data in Fig. 11 it is apparent that 1 µM DCMU raises I_1_ and lowers P to the original I_2_ level, thus eliminating the I_2_-P phase “from above.” Consequently, 1 µM DCMU also suppresses the induction of Fv(I) (Figs. 11b and 12). At the non-saturating concentration of 0.1 µM a distinctly lower Fv(I) amplitude is observed, induction of which is slowed down, with the peak being shifted to longer time (Figs. 11a and 12). Obviously, a common PSI electron acceptor pool fills up more slowly when part of PSII is blocked by DCMU and full reduction of this pool is prevented by light activation of the reactions further downstream of PSI.

These data confirm the previous findings of Neubauer and Schreiber (1987) and are in line with the interpretation that I_2-_P reflects Fv(I), induction of which requires reduction of the PS I acceptor side as well as of the primary donor P700 (Schreiber and Klughammer 2021). By blocking intersystem electron transport at the PS II acceptor side, DCMU prevents both the reduction of P700 by PS II and the reduction of ferredoxin by PS I. The apparent discrepancy with the findings of Toth et al. (2005) calls for clarification by further research. In any case, the presented *Chlorella* data show that the leaf data of Toth et al. (2005) should not be generalized and used as an argument against the existence of Fv(I) *in vivo*.

### Identification of “extra Fv(I)” in a variety of photosynthetic organisms

Measurements of polyphasic rise kinetics were carried out with a large variety of photosynthetic organisms using 720ex and 540ex under similar conditions as outlined above for *Chlorella*. In all these measurements, particular attention was paid to assure that the 720ex and 540ex fluorescence yields are assessed under close to equal conditions, particularly with respect to the effective PAR of the applied actinic illumination (i.e., 540 nm MT). In the case of leaves, mosses, and ferns, satisfactory results were obtained essentially with the same geometry as shown in the block diagram of Fig. 1, when light green, young samples were used. The cuvette was replaced by a 14 × 10 mm frame on which the tissues were fixed with the help of double-sided tape, with this frame being placed in the optical center at 45° angle with respect to the FR-ML and 540 nm ML sources, as well as to the 540 nm actinic LED array and the detector. Leaf samples were positioned such that the adaxial surface (upper side) was directed towards the actinic LED array and 540ex, with the abaxial surface (lower side) facing the 720ex and detector. This geometry generally provided similar O-I_1_ rise kinetics with 720ex and 540ex, which shows that the effective PAR at the sites of fluorescence excitation was close to equal in the two types of measurements.

**Fig. 13:**
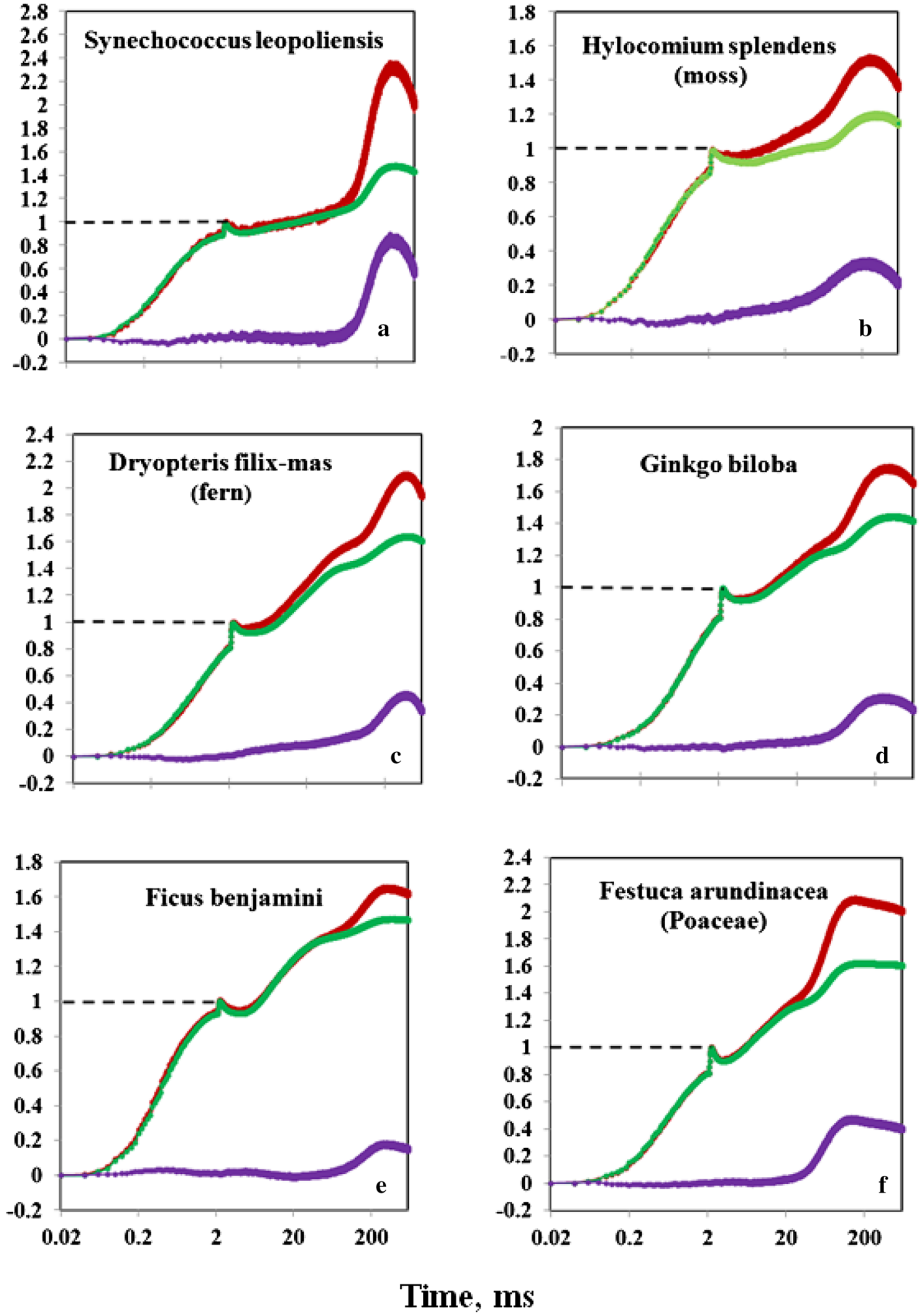
Typical examples of comparative 720ex and 540ex measurements of Fv from a variety of photosynthetic organisms. All recordings were O-I_1_ normalized, so that the difference between the 720ex (dark red) and 540ex (green) responses reflects the “extra Fv(I)” contained in Fv(720ex) compared to Fv(540ex)(violet). Light-green young samples were selected for the measurements in b-f. See text for details on optical geometry

Examples of typical responses observed with cyanobacteria, mosses, ferns, and higher plant leaves are presented in Fig. 13. In all examples, there is substantial “extra Fv(I)” with 720ex compared to 540ex, resulting in distinct peaks of apparent Fv(I) in the 100–200 ms time range. It seems preferable at this stage to refrain from trying to interpret some differences in the detailed features of the various responses between the various species. These examples are meant only to demonstrate the universality of the “extra Fv(I)” phenomenon among photosynthetic organisms. More work will be required to optimize measurements with optically dense and physiologically heterogeneous samples like leaves, mosses, and ferns.

## Discussion

The presented data provide further evidence for the existence of Fv(I) *in vivo*. This evidence relies on five basic assumptions, which are in line with well-established mainstream views:

1. The photosynthetic apparatus consists of the two photosystems, PS I and PS II.
2. Excitation of PS I and PS II gives rise to F(I) and F(II), the excitation and emission spectra of which overlap at room temperature.
3. The ratio of F(I)/F(II) increases at emission wavelengths > 700 nm.
4. The excitation ratio of F(I)/F(II) increases at excitation wavelengths > 680 nm.
5. The O-I_1_ fluorescence rise reflects the light-induced closure of PS II reaction centers and, hence, may serve as a representative measure of Fv(II).

A closer look at the data, however, raises some questions, the answers to which may go beyond some generally accepted views and, hence, may contribute to a new understanding of the relationship between PAM measurements of F(I) and F(II), the distribution of excitation energy between the two photosystems and measurements of PSI/PSII action spectra *in vivo*.

An unexpected new finding is the coexistence of a pronounced O-I_1_ rise transient along with an enhanced I_2_-P transient in the recordings with FR excited fluorescence. Particular attention was paid to the possibility that the measured O-I_1_ is just a consequence of a short-wavelength tail of the FR-ML passing the excitation filter set. Such a tail, however, was not observed by direct analysis of the 720ex, 730ex, and 740ex ML using a Zeiss spectrophotometer. Also, any short-wavelength tail would be expected to decrease using 730ex and 740ex compared to 720ex, thus causing the I_2_-P/O-I_1_ ratio to increase. In reality, however, the I_2_-P/O-I_1_ ratio *decreased* with 730ex and 740ex compared to 720ex (see Fig. 9b). Furthermore, essentially the same O-I_1_/I_2_-P ratio was observed when the FR-ML array was replaced by a 725 nm laser diode, in which case the interference of shorter wavelengths exciting PS II can be excluded (Christof Klughammer, personal communication).

Light-induced changes of FR excited chlorophyll fluorescence have not been measured before, presumably due to severe difficulties in the assessment of relatively small genuine changes of fluorescence against a large background signal caused by scattered FR-ML. It may be pointed out that it would be practically impossible to identify Fv(I) by measuring FR excited fluorescence with standard Chl fluorometers that apply the same light source for fluorescence excitation and actinic illumination, as FR light cannot induce the closure of PS II and PS I reaction centers, which cause the O-I_1_ and I_2_-P transients, respectively.

The finding of close to identical Fv(II) responses in the O-I_1_-I_2_ kinetics using 540ex and 720ex raises the question of how it is possible that the state of PS II reaction centers can be assessed with the help of 720 nm quanta. This question is closely related to the experimental fact that *in vivo* PS II activity driven by FR illumination can be detected at wavelengths up to 780 nm (Pettai et al. 2005) and even 800 nm (Thapper et al. 2009), respectively, and that a fluorescence increase can be measured upon FR illumination (Schansker and Strasser 2005). In Pettai et al. (2005), three different interpretations for FR-driven PSII activity were discussed:

1. Existence of FR-absorbing forms of chlorophyll in PSII.
2. Uphill transfer of excitation energy from PS I to PS II.
3. Absorption from thermally populated vibrational sub-levels of PS II chlorophylls.

Pettai et al. (2005) favored the first interpretation, estimating that their data of FR-driven O_2_ evolution in leaves can be explained by the presence of about one FR-absorbing Chl in each PSII unit. They also estimated that of the absorbed FR quanta 10 × more are distributed to PSI than to PSII. While at that time the presence of “red Chls” *in PSI* already was well established (Gobets and Grondelle 2001), clear-cut evidence for their presence in *PSII* was not yet available. In the meantime, such evidence has been reported for various organisms (for a recent review, see Santabarbara et al. 2020).

As it appears, the “red Chls” (and not the bulk Chl of PSI) are mainly responsible for absorption and fluorescence excitation in the FR spectral range and also for FR fluorescence emission, which should be particularly true for the F > 765 nm measured in the present study. The room temperature emission spectra of the “red Chls” in PSI are very broad (Gobets and van Grondelle 2001; Gobets et al. 2001), consisting of various components with different excitation and emission properties. Their fluorescence lifetimes are distinctly longer than those of the bulk Chls and, therefore, their contribution to steady-state fluorescence (i.e., the time-integrated signal of the multi-phasic fluorescence decay, as measured by PAM fluorometry) is relatively enhanced with respect to bulk fluorescence. In Santabarbara et al. (2020) several reports are cited, in which the presence of “red Chls” in PSII is suggested. While it would go beyond the scope of the present study on Fv(I) to discuss the evidence presented in these reports, it is clear that “red Chls” in the PSII antenna will substantially shift PSII absorption towards longer wavelengths and also increase the lifetime of the excited fluorescence.

If it is assumed that “red Chls” are not only present in PSI, but in the PSII antenna of *Chlorella* as well, the observed O-I_1_ kinetics with 720ex can be readily explained: The excitons formed upon absorption of the pulse-modulated 720 nm quanta by the “red Chls” are thermally activated to carry out charge separation in PSII, so that an increase of pulse-modulated F(II) is indicated when PSII reaction centers close in response to non-modulated actinic illumination. No difference with the kinetics measured with 540ex are expected. For obtaining the same O-I_1_ amplitude with 720ex as with 540ex more 720 nm quanta than 540 nm quanta are required. In practice, however, it does not matter whether equal O-I_1_ amplitudes with 720ex and 540ex are realized via adjusting the ML intensity and the amplifier gain or via normalization. In any case, the PSII responses are made equal and consequently the difference between the 720ex and 540ex responses reflects the excess or “extra Fv(I)” contained in the 720ex compared to the 540ex response. As this “extra Fv(I)” develops in the time range of the I_2_-P transient, it also may be called “extra I_2_-P.”

For an evaluation of the “extra I_2_-P” identified in the present and preceding study, it is important to know whether the whole I_2_-P transient is due to Fv(I) or possibly part of it only. While in the former case, the deconvoluted Fv(II) would not show any significant transient with the “kinetic fingerprint” of the “extra I_2_-P,” in the latter case Fv(II) would display an I_2_-P transient with similar kinetics as the “extra I_2_-P.” For a quantitative deconvolution into the Fv(I) and Fv(II) components, information on the F(I)/F(II) excitation ratio is required. In the present study, this was obtained via the deconvolution of Fo(540ex) and Fo(720ex) into their Fo(I) and Fo(II) components (Figs. 4–7 and supplementary figures S6–S10). Revisiting the data in Fig. 8 of Schreiber and Klughammer (2021), analogous information was also derived by deconvolution of Fo(> 700) and Fo(< 700) (Supplementary Materials, Sect. 6).

### F(I)/F(II) excitation ratios and quantification of Fv(I)

While determination of the “extra I_2_-P” or “extra Fv(I)” in Fv(720ex) can be carried out without any bias or assumptions, assessment of the *total Fv(I)* contained in Fv(720ex) and, hence, quantitative deconvolution into the Fv(I) and Fv(II) components, is possible only either

- by assuming ad hoc that the whole I_2_-P transient is due to Fv(I) or
- by making a plausible assumption on the F(I)/FII) excitation ratio for the given sample under the given conditions.

In practice, possibility (a) is much more convenient than possibility (b) and, therefore, some efforts were made to clarify whether this assumption is realistic. Revisiting the data of Schreiber and Klughammer (2021), in Sect. 6 of Supplementary Materials it is shown that with assumption (a) an apparent excitation ratio of F(I)/F(II) = 35/65 results for F > 700 in *Chlorella* (with 440ex). This ratio is in line with values ranging from 30/70 to 40/60 determined in various C3 photosynthetic organisms (Genty et al. 1990; Pfündel 1998; Franck et al. 2002; Peterson et al. 2014). Notably, assuming the same excitation ratio with 540ex, quite plausible deconvolutions of Fv(I) and Fv(II) kinetics were also obtained for *Chlorella* when F > 765 was measured (see Figs. 6–7 and supplementary figures S6–S9). Hence, the combined evidence presented in Schreiber and Klughammer (2021) and in the present communication strongly argues in favor of the assumption that at least in *Chlorella,* the whole I_2_-P transient is due to Fv(I).

Compared with Fv(II), the amplitude of the observed Fv(I) is relatively small. In *Chlorella*, it amounts to 10–20% of overall Fv. In this context, the differences between the average lifetimes of PSI and PSII fluorescence have to be considered. The product of fluorescence yield and lifetime determines the PAM signal, which is formed by integration of the fluorescence decays during the 1 µs time period of an ML pulse. After dark-adaptation (reaction centers open) the average lifetime of Fo(I) is at least 3 × shorter than that of Fo(II) (see e.g., Chukhutsina et al. 2020). Furthermore, while closure of PSII is known to cause a large increase of the F(II) lifetime (factor of 4–8), so far no increase of the room temperature lifetime of F(I) upon closure of PSI has been reported. This could be mainly due to the difficulty to reproducibly block the PSI acceptor side. As demonstrated in the present study, transient closure of PSI can be achieved *in vivo* during application of saturating multiple turnover pulses of light. The thus induced increase of F(I) intensity (somewhat less than a factor of 2 in *Chlorella*) is much smaller than in the case of F(II). With the average lifetime of Fo(I) *in vivo* amounting to about 80 ps (Chukhutsina et al. 2020) it may be predicted that upon closure of PSI the lifetime of F(I) is increased to about 160 ps.

### Apparently “too large” contribution of F(I) to F < 710 in Schreiber and Klughammer (2021)

Very recently, Pfündel (2021) concluded that “… variable PSI fluorescence can only explain a minor fraction of the wavelength-sensitive part of the I_2_-P phase,” based on comparative measurements of F > 700 and F < 710 with intact leaves of cherry laurel, supporting a similar conclusion of Peterson et al. (2014). A major argument in favor of this conclusion is the apparently “too large” amplitude of I_2_-P in short-wavelength fluorescence. For long time, there has been a general consensus in the literature that short-wavelength fluorescence < 710 nm is mainly due to F(II), while F > 700 is enriched in F(I). If this were true, it would be surprising indeed that the amplitude of I_2_-P in F > 700 is not more than about 1.5 × higher than in F < 710 (see e.g., Fig. 4 in Schreiber and Klughammer 2021). However, relatively recent work has shown that F < 710 may contain distinctly more F(I) than assumed so far: Galka et al.(2012) and Wientjes et al. (2013) first presented evidence for LHCII forming a supercomplex with PSI, so that LHCII has to be considered a constitutive part of the PSI antenna system *in vivo*. In the meantime, this finding has been confirmed and extended by numerous laboratories (Grieco et al. 2015, Bell et al. 2015, Yadav et al. 2017, Bos et al. 2017, Pan et al. 2018, Chukhutsina et al. 2019, Chukhutsina et al. 2020). Hence, with LHCII being part of PSI, F < 710 can no longer be regarded to reflect almost exclusively F(II). This is true for Fv as well as for Fo and has to be taken account of in the evaluation of the “extra Fv(I)” observed in Fv > 700 compared to Fv < 710. It can be said with some certainty that this would be much larger, if LHCII would not bind to PSI. Conversely, measurements of the “extra Fv(I)” may be expected to provide information on the extent of LHCII binding to PSI.

In Supplementary Materials Sect. (6), the *Chlorella* data in Schreiber and Klughammer (2021) are revisited in order to check, whether the factor of 1.5, which was empirically estimated for the ratio of I_2_-P(> 700)/ I_2_-P(< 710), does also hold for the ratio of Fo(I) > 700/Fo(I) < 710. This was found to be true when an excitation ratio F(I)/F(II) = 35/65 was assumed. Hence, similarly as in the case of the F(720ex) vs. F(540ex) data of the present study in Supplementary Materials Sect. (4), also the F > 700 vs F < 710 data in Schreiber and Klughammer (2021) are in line with the notion that the whole I_2_-P phase is due to Fv(I). This conclusion, if confirmed by measurements with other photosynthetic organisms, may prove of considerable practical importance, as it would provide a means for determination of the Fo(I) contained in Fo, based on “extra I_2_-P” measurements and, hence, allow to determine the ratio of F(I)/F(II) excitation *in vivo*.

### Apparently “too small” F(I)/F(II) excitation ratio with 720ex

While the data presented in Figs. 6 and 7 and supplementary figures S6–S10 leave little doubt that in *Chlorella* under the given conditions the ratio of F(I)/F(II) is just 2 × higher with 720ex compared to 540ex, this ratio appears to be “too small.” Based on the information from PSI and PSII action spectra, a 4–5 times higher ratio would appear appropriate. It may be considered a fact that with 720nm light PSI is excited much more than PSII. So why then is Fo(I)720ex not much larger than Fo(I)540ex and I_2_-P(720ex) not much larger than I_2_-P(540ex)? At the present state of information no definite answer to this question can be given. However, it appears likely that any future answer will involve the “red Chls” (for reviews, see e.g., Gobets and van Grondelle 2001; Gobets et al. 2001; Croce and van Amerongen 2013; Krüger et al. 2014; Santabarbara et al. 2020). In this context, it is important to recall that the present study relies on measurements of extremely long-wavelength F > 765, which is *not* determined by the properties of the bulk PSI antenna system. With the “red Chls” being mainly responsible for long-wavelength emission *in vivo*, the measured F(I) and F(II) amplitudes are bound to be effected by the particular properties of the “red Chls,” more of which are present in PSI than in PSII. Various components of F(I) with largely different lifetimes have been identified by decay-associated spectroscopy (DAS) (Slavov et al. 2008; Chukhutsina et al. 2020), with each component reflecting an excitonically separated domain within the PSI antenna system, characterized by different “red Chls”/bulk Chl/RC(I) compositions. It appears reasonable to assume that the F > 765 signal of the present study mainly monitors the “red Chls” emission with the longest lifetime, i.e., of a fraction of pigments that does not contribute much to the effective cross-section of PSI. On the other hand, in the case of PSI and PSII action spectra, the properties of the *bulk pigments* are decisive, which absorb most of the incident actinic light. In principle, the apparently “too small” F(I)/F(II) excitation ratio with 720ex could indicate that excitons from quanta absorption by “red Chls” are more likely to be non-photochemically quenched than excitons from absorption by bulk Chls. This would result in a relatively low photochemical yield of 720 nm quanta absorbed by “red Chls” in PSI, without significantly affecting the known high PSI quantum yield, as the ratio of “red Chls”/bulk Chls is low. However, other explanations cannot be ruled out.

Grieco et al. (2015) identified a large excess of LHCII not present in the isolatable PSII-LHCII and PSI-LHCII complexes of *Arabidopsis*, concluding that their data provide “evidence that PS I and PS II are traps embedded in the same energetically connected LHCII lake.” Yokono et al. (2015) reported that about half of PSII is physically connected to PSI in *Arabidopsis*, as concluded from the properties of a soluble megacomplex composed of PSI, LHCI, PSII, and LHCII. Provided that these reports describe correctly the physiological reality *in vivo*, excitation energy could efficiently equilibrate between PSI and PSII in a common domain of the thylakoid membrane, which would have far-reaching consequences for the interpretation of Chl fluorescence in general. The fluorescence emitted from the common domain would respond to changes of both PSI and PSII, so that it would be expected to display changes that differ from those of F(I) and F(II) in the excitonically separated PSI and PSII domains. In an envisaged 3-domain model, the Fv(I) identified in Schreiber and Klughammer (2021) and the present study would be likely to originate from a separate type of PSI that is excitonically segregated.

As was pointed out by Trissl and Wilhelm (1993), a close proximity between PSI and PSII would bear the risk that PSI drains off excitation energy from PSII, mainly because of its faster trapping kinetics. Therefore, a common PSI and PSII domain appears feasible only in conjunction with an effective “switch” that regulates the distribution of excitons between PSI and PSII (Clayton 1963). It is tempting to speculate that the conformational LHC switching discovered by Krüger et al. (2011) could play a regulatory role in exciton distribution. In the classical bipartite model of Butler and Kitajima (1975) (see also review in Butler 1978), energy transfer from PSII to PSI by “spillover” was thought to be responsible for the parallel light-induced increases of F730 and F690 at 77 K in chloroplasts. If spillover contact between PSII and PSI would also exist *in vivo*, it would enable equilibration of excitons between the two photosystems, so that a spectral distinction of F(I) and F(II) in the fluorescence originating from this “mixed domain” would not be possible at room temperature. Presumably, there would also be “reverse spillover,” i.e., up-hill transfer of excitation energy from PSI to PSII, with the deficit energy provided by phonons. In principle, the “mixed domain” fluorescence could respond to changes in photochemical efficiency of both photosystems. But, due to the distinctly faster trapping kinetics in PSI and non-photochemical quenching by P700^+^, the PSII responses would be expected to be much more pronounced.

The above considerations give an impression of the complexity of parameters that have to be taken into account in the evaluation of the relative amplitudes of Fv(I) measured with 720ex and 540ex. A definite explanation of the surprisingly low excitation ratio of F(I)/F(II) with 720ex must await further progress in the elucidation of the role of the “red Chls” and in the identification of a putative “mixed domain” of PSI and PSII *in vivo*. Conversely, the properties of Fv(I) may help to investigate the regulatory function of the “red Chls” *in vivo* and to obtain evidence for the existence of megacomplexes of PSI, PSII, LHCI, and LHCII *in vivo*. In this context, it may be pointed out that the Fv(I) measured by PAM fluorometry carries the “kinetic fingerprint” of the changes in energy conversion in PSI, which in addition to the spectral properties can serve as a “marker” for PSI fluorescence.

### Significance of Fv(I) for the interpretation of the polyphasic rise kinetics

Until recently, the polyphasic rise kinetics of fluorescence yield upon the onset of strong actinic light have been interpreted almost exclusively in terms of Fv(II) (for reviews see e.g., Schreiber 2004; Strasser et al. 2004; Lazar 2006; Stirbet and Govindjee 2012; Schansker et al. 2014). While there has been general consensus that the “photochemical” O-I_1_ (or O-J) phase reflects Fv(II) upon closure of PSII reaction centers, quite different interpretations have been proposed for the “thermal” I_1_-I_2_-P (or J-I-P) phases. With the new evidence for I_2_-P reflecting Fv(I) in *Chlorella*, previous interpretations of this part of the polyphasic rise kinetics have to be reconsidered. As to the preceding I_1_-I_2_ phase, the data presented here and in Schreiber and Klughammer (2021) clearly show that it reflects Fv(II), which raises the question of the nature of quenching at I_1_ with respect to I_2_. Numerous mechanisms involving the PSII acceptor and donor sides, oxidized PQ, as well as “conformational” changes at the level of PSII reaction centers, have been discussed in the literature. A key observation is the elimination of this quenching by DCMU (Neubauer and Schreiber 1987; Samson and Bruce 1996; Schreiber 2002, 2004; Prasil et al. 2018). Supporting earlier conclusions of Schansker et al. (2011), a quenching mechanism based on a PSII conformational change was favored by Magyar et al. (2018) and Sipka et al. (2021), based on measurements of the flash-induced fluorescence rise in the presence of DCMU. In these studies, an intermediate fluorescence yield F1 was reached after a saturating 1.5 µs flash and the maximal fluorescence yield Fm could be reached with a large number of such flashes only. The transition from F1 to Fm was considered to reflect a photochemically induced protein conformational change that “is responsible for a large part of Fv” *in vivo* (Sipka et al. 2021). In view of the results of the present study, a contribution of this kind of Fv to the overall increase from Fo to Fm (or P) appears feasible for the I_1_-I_2_ phase only. This, however, cannot only be eliminated by DCMU (Fig. 11), but also largely suppressed by pre-reduction of the PQ pool in the dark. In Fig. 8c the amplitudes of P, I_1_, and the “extra Fv(I)” amount to 1.63, 1.00, and 0.31, respectively. Assuming that in *Chlorella* Fv(I) = 2x “extra Fv(I)” almost no I_1_-I_2_ remains that possibly could be associated with an Fv due to a light-driven conformational change. It cannot be excluded, however, that a conformational change is induced when the PQ pool becomes reduced in the dark. Any further attempt to give an answer to the origin of the quenching at I_1_ and of the I_1_-I_2_ phase would be beyond the scope of the present communication.

### Consequences of the existence of Fv(I) on quenching analysis

In practical applications, saturation pulse quenching analysis has been of particular value, because it allows the assessment of the photosynthetic state of a sample *in a given state of illumination*. Under these conditions, where the reactions downstream of Fd are light-activated, the interference of Fv(I) is minimized (see e.g., Fig. 6 in Schreiber and Klughammer 2021). Special attention is indicated, however, when maximal Fv/Fm is measured after dark-adaptation, where the values of Fv and Fm contain a substantial I_2_-P component and, hence, also a significant amount of Fv(I). This problem can be avoided by replacing strict dark-adaptation by adaptation to low-intensity background illumination. The preillumination should activate the reactions downstream of Fd, without causing significant energy-dependent non-photochemical quenching, NPQ. The resulting suppression of I_2_-P unavoidably results in lower values of Fv/Fm. On the other hand, higher values of Fv/Fm are obtained, when the contribution of F(I) to Fo is corrected for.

When the existence of Fv(I) is ignored, this may lead to misinterpretations of light-induced fluorescence changes. For example, non-photochemical quenching (NPQ) normally is quantified with reference to the Fm state measured after dark-adaptation. When upon illumination at moderate light intensity the maximal fluorescence yield Fm’ declines relative to Fm, this formally leads to an increase of NPQ, whereas in fact the decline of Fm’ is due to suppression of the I_2_-P phase by light activation of the reactions downstream of PSI. This aspect should be accounted for in quantitative studies of NPQ by taking the I_2_ level after dark-adaptation instead of the Fm level as reference (as originally suggested in Schreiber et al. 1995).

### Why was Fv(I) not discovered much earlier?

When variable Chl fluorescence was discovered more than 90 years ago by Hans Kautsky (Kautsky and Hirsch 1931), no information on the existence of two photosystems was available yet. Such information gradually emerged 30 years later and Chl fluorescence proved a pioneering tool in the elucidation of the various electron transport steps between the splitting of water in PSII and the reduction of NADP at the acceptor side of PSI (for an early review, see e.g., Lavorel and Etienne 1977). While it was clear that part of fluorescence originates from PSI, this was thought to be constant, contributing to the dark fluorescence, Fo, only. As we know now, the amplitude of Fv(I) indeed is quite small compared to Fv(II). Furthermore, Fv(I) develops *transiently* only upon illumination with saturating light in a relatively short time window (between 20 and 200 ms). Last, but not least, Fv(I) is suppressed upon continuous illumination and in the presence of DCMU (see Figs. 11 and 12). While DCMU has been extremely valuable in the study of PSII primary reactions by blocking secondary electron transport, an analogous inhibitor does not exist for PSI. Presumably, this is the main reason why so far Fv(I) has not been detected by powerful time-resolved fluorescence measuring techniques, which during the past 30 years have been applied in numerous studies on the fate of excitation energy absorbed in the two photosystems.

In principle, decay-associated-spectroscopy (DAS) allows a detailed and accurate analysis of the properties of Chl fluorescence *in vivo* (for impressive recent reports see Wientjes et al. 2017, Chukutsina et al. 2019; Chukhutsina et al. 2020). PSI and PSII fluorescence contributions can be readily differentiated based on their vastly different lifetimes and decay-associated excitation and emission spectra. When, however, as common practice for detection of Fv(II), the PSII inhibitor DCMU is added, Fv(I) is effectively prevented (Figs. 11 and 12). Induction of Fv(I) is also prevented when the reactions downstream of PSI are light-activated by preillumination (see e.g., Fig. 6 in Schreiber and Klughammer 2021). After dark-adaptation, a very strong multiple turnover light pulse must be applied to assure that the bottleneck at the acceptor side of PSI (ferredoxin) is closed, before it is opened again by light activation, which is particularly fast in algae and cyanobacteria (see responses in Fig. 13). On the other hand, as it may be expected that the lifetime of Fv(I) is considerably longer than that of Fo(I) and as it should be considerably red shifted with respect to Fv(II), confirmation of its existence by time-resolved measurements should not be too difficult, when the above requirements are fulfilled.

## Summary and conclusions

1. A Multi-Color-PAM fluorometer was extended for measurements of dark–light Chl fluorescence induction kinetics using FR pulse-modulated measuring light.
2. Using this measuring system, for the first time the polyphasic rise kinetics with prevailing excitation of PSI fluorescence, F(I), was measured at wavelengths > 765 nm.
3. A relatively large constant background signal contained in the original 720ex signal was quantified and corrected for, so that not only quantitative information on Fv(I), but also on Fo(I) became available.
4. Following an approach introduced by Schreiber and Klughammer (2021), the 720ex and 540ex responses were rescaled such that their O-I_1_ amplitudes were equal and, hence, all F(II) responses were equalized.
5. From the difference between the O-I_1_ equalized Fv(720ex) and Fv(540ex) responses, a selective Fv(I) response was derived, corresponding to the “extra Fv(I)” observed with 720ex compared to 540ex.
6. Analysis of the Fo(I) and Fo(II) contributions to Fo(720ex) and Fo(540ex) revealed that in dark-adapted *Chlorella* the ratio of Fo(I)/Fo(II) with 720ex exceeds that with 540ex by a factor of 2, when a plausible ratio of Fo(I)/Fo(II) = 35/65 with 540ex is assumed.
7. Assuming that also the ratio of Fv(I)/Fv(II) with 720ex exceeds that with 540ex by a factor of 2, it was deduced that in dark-adapted *Chlorella* the Fv(I) contained in Fv(720ex) amounts to 2 × the “extra Fv(I).”
8. Based on this Fv(I) information, the Fv(II) kinetics were deconvoluted, which closely match the original Fv(720ex) kinetics up to about 20 ms, where Fv(I) starts developing. Thereafter, saturation of Fv(II) sets in, while Fv(I) displays a transient peak at about 200 ms. The data support the notion that the I_2_-P (or I-P) phase is due to Fv(I).
9. As in *Chorella* depending on experimental conditions a distinct I_2_ step or inflection cannot always be distinguished, the I_2_ level was defined such that the difference between the P and I_2_ levels equals the Fv(I) amplitude, i.e., twice the measured “extra Fv(I).”
10. Comparative measurements of dark–light induction kinetics with 720ex and 540ex at lower actinic intensities revealed that some Fv(I) is also hidden in the peak of the “classical” Kautsky effect (O-I-P-S transients), when no I_1_ and I_2_ levels can be distinguished.
11. DCMU was found to suppress the I_2_-P phase “from above,” in line with the conclusion that maximal F(II) is indicated by I_2_ and not by P (or Fm).
12. Comparative measurements with 540ex and various excitation wavelengths in the 680-740 nm range revealed that the “extra Fv(I)” develops at wavelengths > 680 nm and reaches a peak at about 720 nm.
13. While the development of Fv(I) in the “red drop” spectral range qualitatively agreed with the well-known increase of PSI/PSII excitation derived from PSI and PSII action spectra, an apparent discrepancy was noted between the observed factor of 2 in the ratio of F(I)/F(II) excitation and a factor of 8–10 in effective PSI/PSII activity. A likely role of “red Chls” in determining the properties of F(I) and F(II) measured at wavelengths > 765 nm is suggested.
14. The F > 700 versus F < 710 data in Schreiber and Klughammer (2021) were revisited, where a relatively large I_2_-P amplitude in the F < 710 response appears to reflect “too much” F(I) < 710. A refined analysis of these data shows that Fo(I) > 700/Fo(I) < 710 = Fv(I) > 700/Fv(I) < 710 = 1.48, when an excitation ratio of F(I)/F(II) = 35/65 for F < 710 is assumed. It is concluded that F < 710 *in vivo* indeed contains more F(I) than commonly assumed and that this is in line with the reports published by a number of laboratories during the past 10 years that LHCII is a constitutive part of the PSI antenna system.
15. Comparative measurements with 720ex and 540ex on a variety of different photosynthetic organisms revealed substantial “extra Fv(I)” in all these organisms. A particularly large “extra Fv(I)” (almost as large as the O-I_1_ phase) was found in cyanobacteria which thus appear particularly well suited for further research on Fv(I) *in vivo*.

## Supplementary Information

Below is the link to the electronic supplementary material.

Supplementary file1 (PDF 603 kb)

## Data Availability

Original data will be provided by the author on request.

## Abbreviations

540ex: 540 nm pulse-modulated light applied for measuring fluorescence yield
720ex: 720 nm pulse-modulated light applied for measuring fluorescence yield
AL: Actinic light
Chl: Chlorophyll
F > 700: Fluorescence detected with detector filter set passing wavelengths > 700 nm
F < 710: Fluorescence detected with detector filter set passing wavelengths < 710 nm
F > 765: Fluorescence detected with detector filter passing wavelengths > 765 nm
Fo, Fm: Minimum and maximum fluorescence yield of dark-adapted sample
F(I), F(II): Fluorescence emitted from PS I and PS II pigment systems, respectively
FR: Far-red light, more strongly absorbed by PSI than by PSII
Fv: Variable fluorescence
Fv(I), Fv(II): Variable fluorescence emitted from PS I and PS II pigment systems, respectively
Fv/Fm: Fluorescence based expression for maximal PS II quantum yield
LED: Light-emitting diode
ML: Pulse-modulated fluorescence measuring light
MT: Saturating multiple turnover light pulse
NPQ: Non-photochemical quenching of chlorophyll fluorescence
O-I_1_-I_2_-P: Characteristic levels of fluorescence yield during the course of the polyphasic fluorescence rise upon transition from the dark to the strongly illuminated state
PAM: Pulse Amplitude Modulation
PAR: Quantum flux density of photosynthetically active radiation
PS: Photosystem
RC: Photosynthetic reaction center
