# Supplementary material for: Light-induced changes of far-red excited chlorophyll fluorescence: further evidence for variable fluorescence of photosystem I in vivo
Source: Photosynth Res. 2023 Jan 4;155(3):247–70. doi: 10.1007/s11120-022-00994-9 (PMC9958156; doi:10.1007/s11120-022-00994-9)
Supplement: Supplementary file 1 — Supplementary file1 (PDF 603 kb) [file 11120_2022_994_MOESM1_ESM.pdf]

Schreiber (2023) Supplementary Materials

(1) Estimation of the PS I/PS II excitation ratio from PS I and PS II action spectra in Schreiber and Vidaver (1974).

Action spectra for PS I and PS II were measured by Schreiber and Vidaver (1974) with suspensions of the unicellular green alga *Scenedesmus obliquus*, the photosynthetic properties of which are similar to those of the unicellular green alga *Chlorella vulgaris* used in the present study. In figure S1below the Fig.7 of Schreiber and Vidaver (1974) is reproduced.

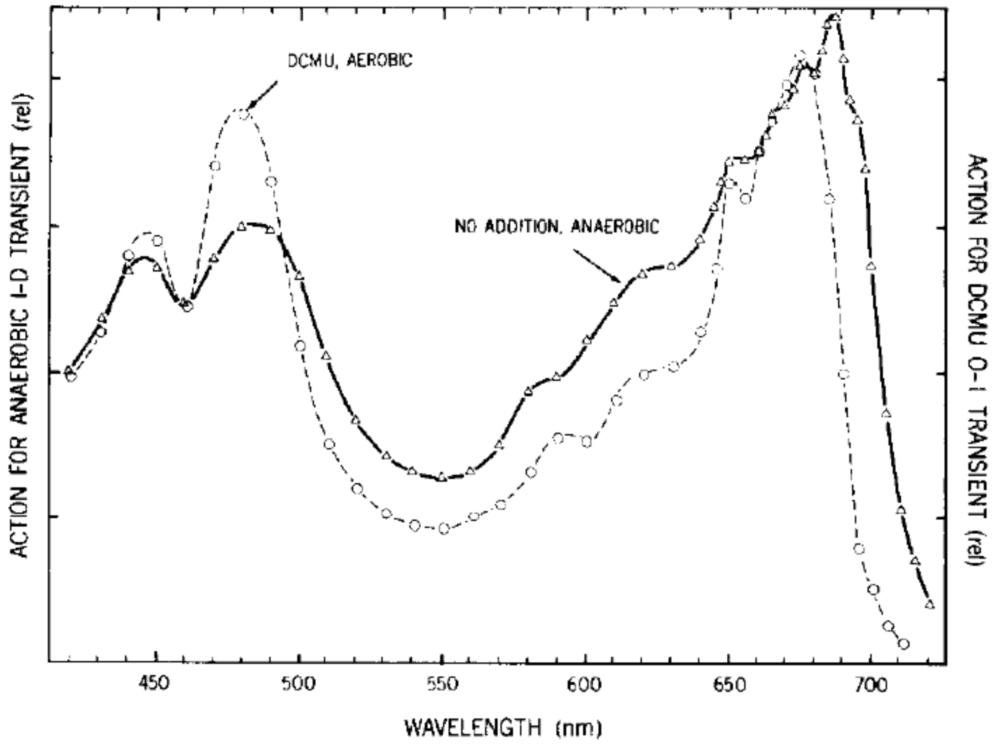

S1 Data from Schreiber and Vidaver (1974) BBA 368:97-112. PSII and PSI action spectra measured with *Scenedesmus obliquus*. PSII action, rate of light-induced fluorescence rise in the presence of 50µM DCMU. PSI action, rate of light-induced fluorescence decay under anaerobic conditions. The curves are normalized at 680nm.

Between 660 and 680 nm the actions of PS I and PS II show close to equal increases. Above 680 nm, however, the PS I action rises to a peak around 690 nm, whereas the PS II action displays a steep drop. A similar drop is observed in PS I action above 690 nm. The data in the red-FR wavelength range (660-715 nm) are replotted in figure S2a and the derived ratio spectrum of in vivo PS I/PS II action is presented in figure S2b. In the context of the present study, the excitation ratio in the “red drop” region between 680 and 715 nm is of particular interest.

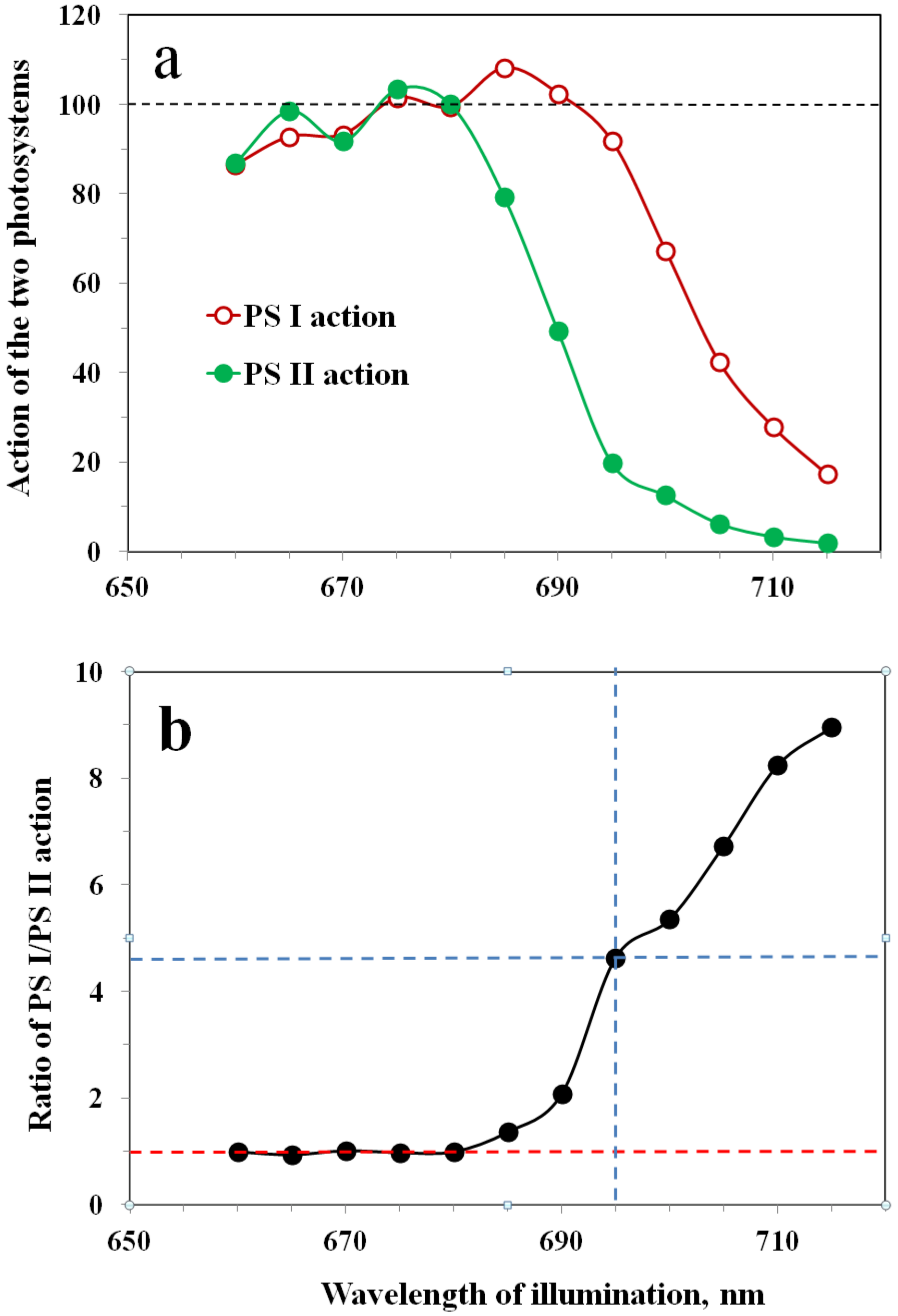

S2 In panel **a** the data between 660 and 715nm of Schreiber and Vidaver (1974) are replotted. The PS II value for 715 nm was estimated by extrapolation. Panel **b** shows the derived ratio spectrum of PS I/PS II action.

Above 680nm the ratio of PSI/PSII action increases in two approximately equal steps, with the 1. step ending at about 695nm, i.e. where PSI action is still high (92%), whereas PSII action has already dropped to 20%. During the 2. step both the PSI and the PSII actions decline, but PSII action more steeply than PSI action.

(2) Estimation of the effective background signal and of Fo in measurements of 720nm excited fluorescence under the conditions of the experiment of Fig.4 described in the main text

Measurements of 720nm excited fluorescence changes were carried out in the optical geometry described in Figs. 1-3 (main text). Due to the weak signals of 720nm excited chlorophyll fluorescence >765nm (720ex), high ML intensity and gain settings had to be applied with 720ex to obtain similar signal amplitudes as with 540ex, for which low ML intensity and gain settings were used. Therefore, the unavoidable background signal (i.e. the signal not consisting of chlorophyll fluorescence) was much larger with 720ex than with 540ex. While in the case of 540ex the background signal was negligibly low, it contributed significantly to the overall signal with 720ex, thus complicating quantitative assessment of Fo(720ex). The following measurements served to estimate the background signal with 720ex under the conditions of Fig. 4 (main text), in order to quantify Fo(720ex) (see also Materials and methods). The obtained Fo(720ex) can be compared with Fo(540ex) and consequently estimates of Fo(I) and Fo(II) may be derived (figures S6 and S7 below).

The background signal with 720ex was composed of optical and non-optical components. The latter consisted of an electrical “pick-up” signal that could be readily determined by shielding the detector (black cardboard in front of photodiode), amounting to 145mV. The optical background signal was heterogeneous, one part being due to 720nm ML reflected from the cuvette walls and another part due to 720nm ML scattered by the *Chlorella* cells towards the detector. The reflectance part was revealed by a “blank” measurement (cuvette filled with suspension medium, without *Chlorella*): The overall blank signal amounted to 270mV, being composed of the 145mV electrical pick-up signal and the reflectance signal. For estimation of the fluorescence signal caused by scattered 720nm ML, first the amount of freshly precipitated $BaSO_4$ was determined that was required to obtain the same scattering signal as with the *Chlorella* suspension (dotted green line in figure S3). For this purpose, the RG780 filter in front of the detector was replaced by a set of neutral density filters attenuating the signal by a factor of 800. 5µl aliquots of a 50mM $BaCl_2$ solution were added to the cuvette filled with 1300µl BG11 suspension medium enriched with 2mM SO_4^{2-}. 25µl of the $BaCl_2$ solution gave the same scattering signal as the *Chlorella* suspension.

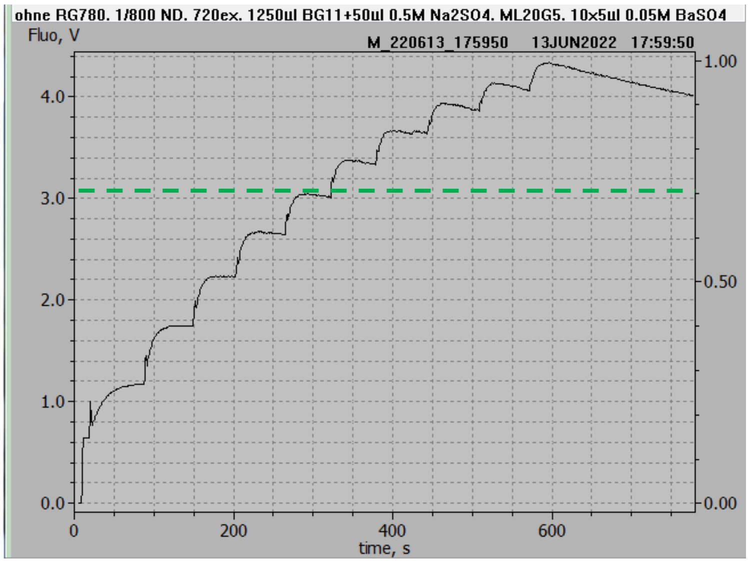

S3 Titration of 720nm scattering signal induced by stepwise precipitation of $BaSO_4$ in the suspension medium. The broken green line indicates the scattering signal measured under the same conditions with a suspension of *Chlorella*, as used in the experiment of Fig.4 (main text). Measurement carried out in the optical geometry described in Fig.1 (main text), with the RG780 filter in front of the detector being replaced by a set of neutral density filters attenuating the signal by a factor of 800. 5µl aliquots of a 50mM $BaCl_2$ solution were added to the cuvette filled with 1300µl BG11 suspension medium enriched with 2mM SO_4^{2-}. After addition of 25µl of the $BaCl_2$ solution a similar signal was reached as with the *Chlorella* suspension.

The supplementary figure S4 shows the increase of the background fluorescence signal upon addition of 25µl $BaCl_2$ solution, as measured in presence of the RG780 filter at the same sensitivity as used in the experiment of Fig. 4 (main text) with the *Chlorella* suspension.

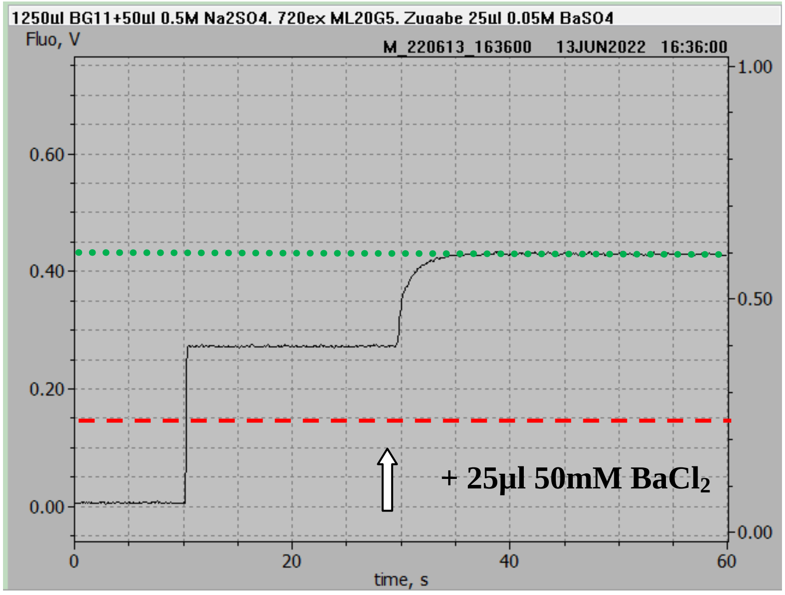

S4 Increase of apparent fluorescence signal of cuvette filled with 1300µl BG11 suspension medium enriched with 2mM SO_4^{2-} upon addition of 25µl of a 50mM $BaCl_2$ solution. 720nm ML switched on at 10s. Optical geometry as described in Fig. 1 (main text), with 3mm RG780 filter in front of detector. The broken red line indicates the contribution of an electrical offset to the overall signal. The signal measured before addition of $BaCl_2$ is composed of the electrical offset and reflectance signals. The overall background signal measured after addition of 25µl $BaCl_2$ solution amounts to 430mV (green dotted line).

It appears reasonable to assume that a similar overall background signal as obtained in presence of freshly precipitated $BaSO_4$ in the experiment of figure S4 (i.e. approximately 430mV) does also apply to the *Chlorella* data in Fig.4 (main text). While the non-optical components *per se* are identical, the scattering components were made equal via the $BaSO_4$ titration and the reflectance components may be assumed to be close to equal, as the color of the *Chlorella* cells should not affect the reflectance of wavelengths >765nm.

The thus estimated background signal can be subtracted from the 720ex response measured with *Chlorella* so that a plausible estimate for the dark fluorescence level, Fo(720ex) = 1000mV can be derived, as shown in figure S5.

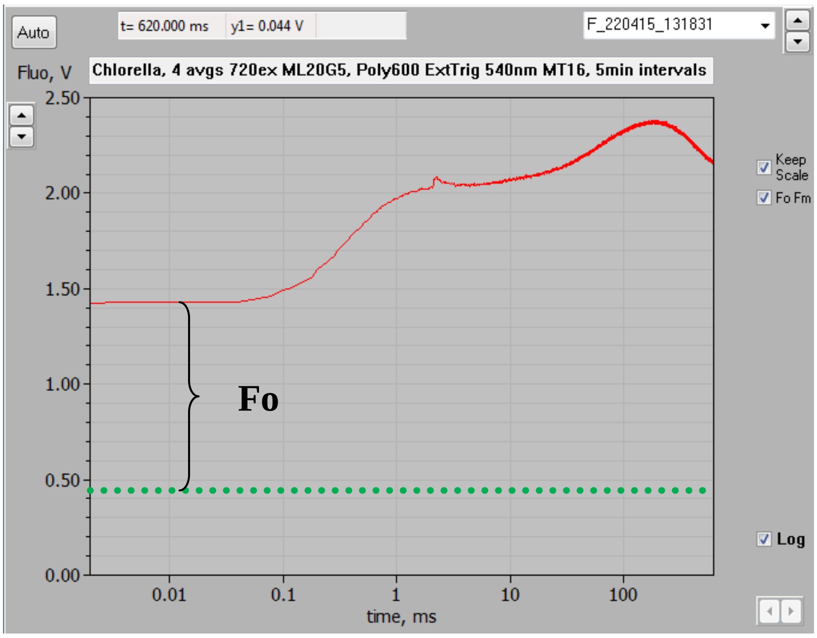

S5 Polyphasic fluorescence rise upon onset of strong actinic illumination measured with dark-adapted *Chlorella* using 720nm pulse-modulated excitation (as also shown in Fig.4, main text) under the same conditions as the measurement of the background signal in figure S4. The green dotted line indicates the corrected baseline, accounting for the background signal estimated in the experiment of S4. The corrected Fo amplitude is shown. This Fo, which amounts to 1000mV, is composed of contributions of Fo(I)720ex and Fo(II)720ex.

(3) Estimation of the Fo(I) and Fo(II) contributions in the 720ex and 540ex responses depicted in Fig. 4 (main text)

The initial fluorescence yield, Fo, in green C3 photosynthetic organisms may be assumed to be composed of about 35% Fo(I) and 65% Fo(II), when fluorescence is excited with visible light and measured at wavelengths >700nm. In figure S6 below, this information is applied to the 540ex data of Fig.4 (main text). At the given ordinate scaling, the resulting Fo(I) and Fo(II) values with 540ex amount to 260mV and 480mV, respectively.

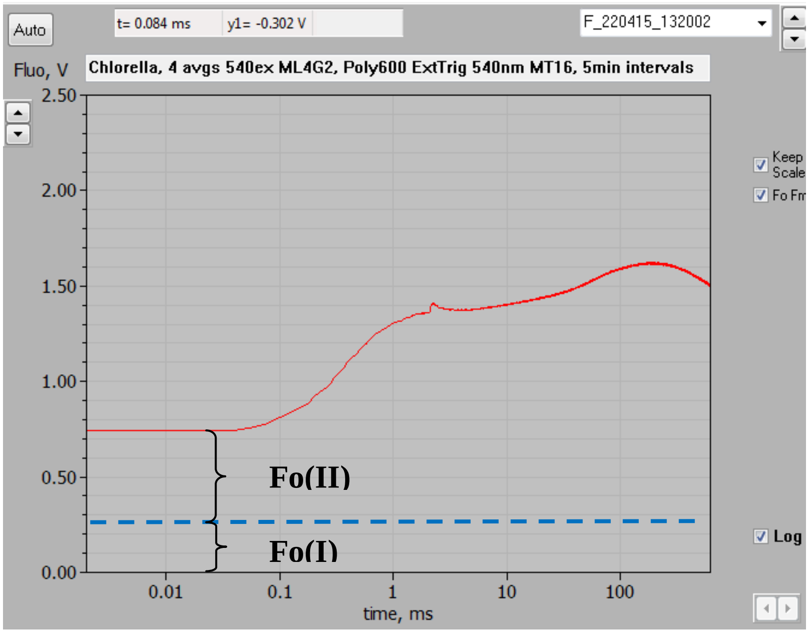

S6 Polyphasic fluorescence rise upon onset of strong actinic illumination measured with dark-adapted *Chlorella* using 540nm pulse-modulated (540ex response of Fig.4, main text). The overall Fo(540ex) of 740mV is assumed to be composed of 35% Fo(I) and 65% Fo(II), i.e. to consist of 260mV Fo(I)540ex and 480mV Fo(II)540ex.

As outlined above (figures S3-S5), in the case of 720ex, quantitative determination of Fo is complicated by an unavoidable, relatively large background signal, for which, however, a plausible estimate could be derived under the conditions of the measurements in Fig.4 (main text). After correction for this background signal, the resulting Fo(720ex) can be readily deconvoluted into its Fo(I) and Fo(II) components. For this purpose, it may be assumed that because of O-I_1 equalization of the 720ex and 540ex responses, Fo(II)720ex is equal to Fo(II)540ex, based on the rationale that O-I_1 is a specific PSII response and that when the O-I_1 amplitudes are equal also the amplitudes of all other PSII responses, including Fo, should be equal. The resulting deconvolution of Fo(720ex) is shown in figure S7.

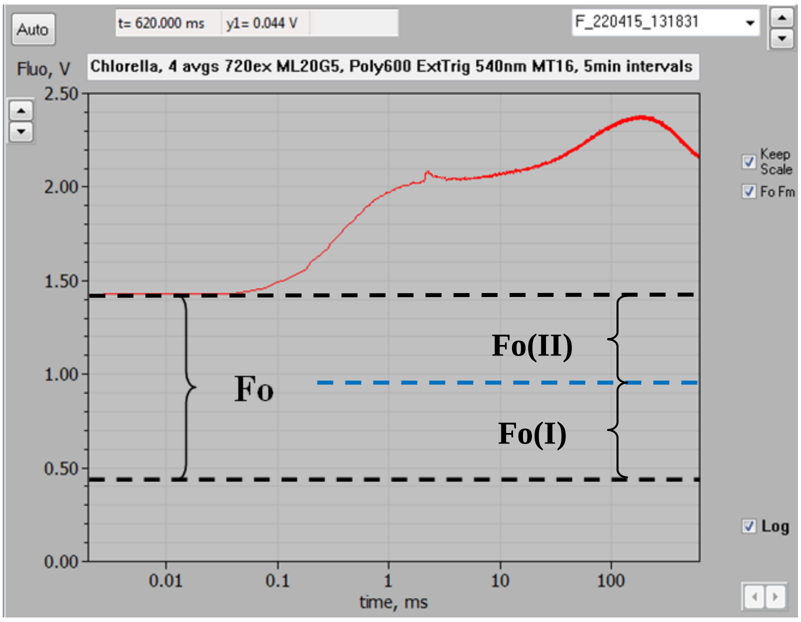

S7 Polyphasic fluorescence rise upon onset of strong actinic illumination measured with dark-adapted *Chlorella* using 720nm pulse-modulated excitation, as also shown in figure S5, with deconvolution of the corrected Fo into the contributions of Fo(I) and Fo(II), based on the assumption that Fo(II)720ex = Fo(II)540ex = 480mV (see figure S6).

With overall Fo(720ex) amounting to 1000mV and Fo(II)720ex = Fo(II)540ex = 480mV, it follows that Fo(I)720ex = 520mV, which happens to be twice the amplitude of Fo(I) 540ex = 260mV in figure S6. As due to the O-I_1 equalization F(II) excitation is equal, this means that with 720ex two times more F(I) is excited compared with 540ex, i.e. F(I)720ex/F(I)540ex = 2, which holds for both Fo(I) and Fv(I). It should be noted that the numerical value of 2 for this excitation ratio relies on the tentative assumption that Fo(540ex)>765nm in *Chlorella* under the given conditions contains 35% Fo(I). While the exact excitation ratio is not known, in the following section the possible influence of variations of this ratio on deconvolution of Fv(I) and Fv(II) is investigated.

(4) Considering variations in the F(I)/F(II) excitation ratio with 540ex

When O-I_1-equalized responses with 720ex and 540ex are analyzed, as in Figs. 6-7 (main text), the excitation ratio F(I)720ex/F(I)540ex corresponds to the factor by which the difference signal Fv(720ex) – Fv(540ex), i.e. the “extra Fv(I)”, has to be multiplied in order to obtain Fv(I)720ex. This factor depends on the assumed F(I)/F(II) excitation ratio with 540ex, which determines the Fo(I)540ex/Fo(540ex). In figure S8 a plot of F(I)720ex/F(I)540ex *versus* the assumed Fo(I) contribution to the total Fo(540ex) is presented for the same original data as in figures S5-S7 (and Fig.4, main text). It can be shown that for *Chlorella* under the given conditions the function y = 0.351/x + 1 applies, derivation of which is based on the following experimental values, definitions and equations, where x is the fraction of Fo(I) in Fo(540ex):

(1) Fo(540ex) = 740 = Fo(I)540ex + Fo(II)540ex

(2) Fo(I)540ex = x*740   Fo(II)540ex = (1-x)*740

(3) Fo(720ex) = 1000 = Fo(I)720ex + Fo(II)720ex

(4) Fo(I)720ex = z*1000 Fo(II)720ex = (1-z)*1000

(5) Fo(II)540ex = Fo(II)720ex (valid after O-I_1 equalization)

(6) (1-x)*740 = (1-z)*1000   z = 0.26 + x*0.74

(7) Fo(I)720ex = z*1000 = (0.26 + x*0.74)*1000

(8) y = Fo(I)720ex/Fo(I)540ex

(9) y = [(0.26 + x*0.74)*1000]/x*740 = 0.351/x + 1

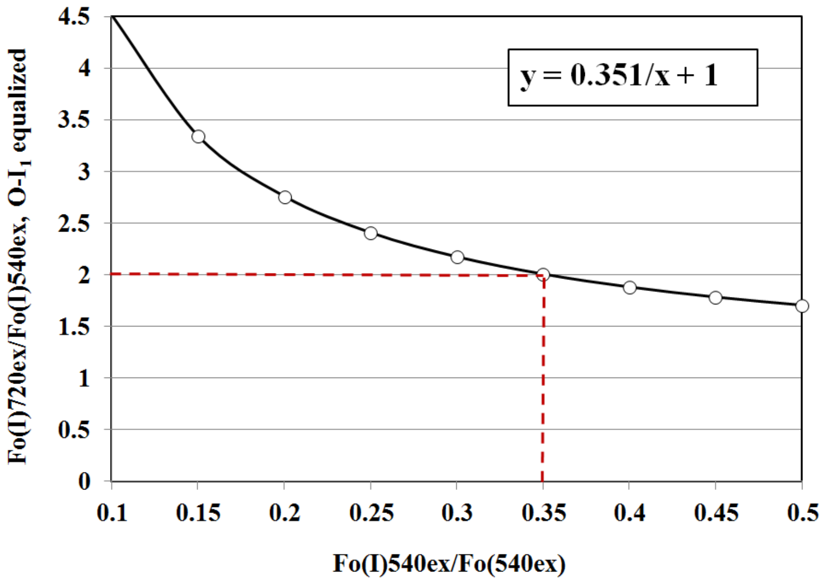

S8 Relationship between the excitation ratio Fo(I)720ex/Fo(I)540ex and the assumed contribution of Fo(I) to total Fo(540ex) in *Chlorella* under the experimental conditions of Fig.4 (main text). In the main text it is assumed that Fo(I)540ex/total Fo(540) = 0.35, so that Fo(I)720ex/Fo(I)540ex = 2. Consequently, the same ratio also applies to Fv(I)720ex/Fv(I)540ex.

The thus defined y-parameter corresponds to the factor by which excitation of F(I) with 720ex exceeds that with 540ex. This not only holds for Fo(I), but for Fv(I) as well. Of central importance is the assumption of Fo(II)540ex = Fo(II)720ex, which is justified when the 720ex and 540ex responses are O-I_1 equalized.

When, as in the case of Figs. 6 and 7 (main text), information on Fo(I) and Fo(II) with 540ex and 720ex is available, this can be applied for deconvolution of the Fv(I) and Fv(II) kinetics in the Fv(720ex) response. The resulting kinetics depend on the assumed value of Fo(I)540ex/Fo(540ex) (i.e. value of x in figure S8), as demonstrated in figure S9 for the data of Fig.7b (main text). Values

of 0.45 (panel a), 0.35 (panel b) and 0.20 (panel c) are assumed, which correspond to excitation ratios Fo(I)720ex/Fo(I)540ex (i.e. y-values in figure S8) of 1.78, 2.00 and 2.76, respectively.

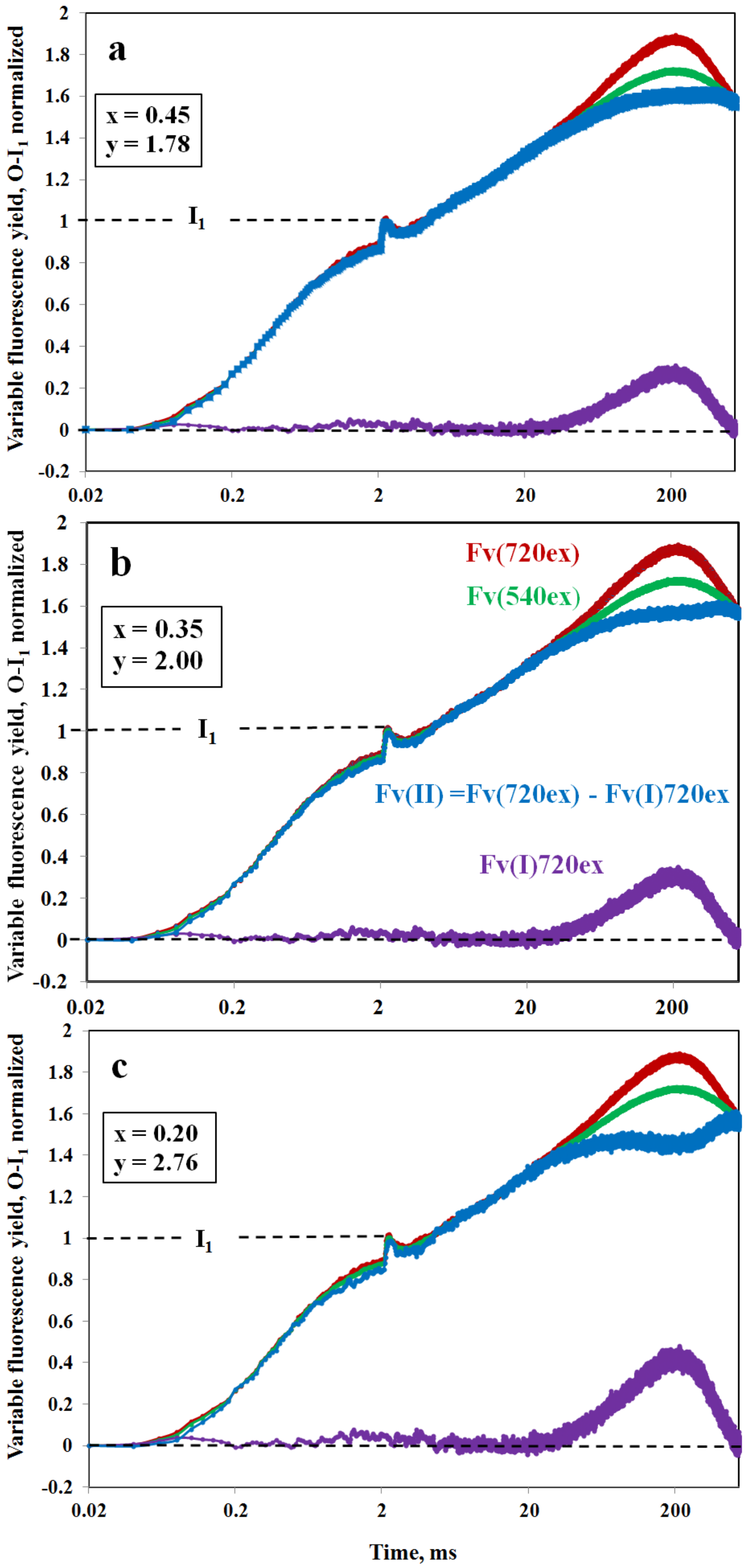

S9 Influence of assumed value of Fo(I)540ex/Fo(540ex) = x on deconvolution of Fv(II). Panel b corresponds to Fig.7b (main text), where it was assumed that Fo(540ex) contains 35% Fo(I).

The data in figure S9 provide information on the extent of variability in deconvolution of Fv(I) and Fv(II) that may be expected depending on the assumed F(I)/F(II) excitation ratio with 540ex. When x = 0.45 is assumed instead of x = 0.35 (used in main text), this leads to a small decrease of Fv(I)720ex and a correspondingly small increase of Fv(II), without significant changes in the Fv(I) anf Fv(II) kinetics. Hence, variations of x in this order of magnitude do not seriously affect the interpretation of the data. On the other hand, assuming x = 0.20 results in substantially higher Fv(I) (by about 30%), leading to a "trough" phase in Fv(II) that is followed by an additional rise phase in parallel with the decline of Fv(I). At the present state of knowledge, such kinetics of Fv(II) seem unlikely.

(5) Deconvolution of the Fv(I) and Fv(II) components in the 540ex response

In Fig.6a (main text) deconvolution of Fv(720ex) into its Fv(I) and Fv(II) components was based on the assumption that Fo(I)540ex contributes 35% to total Fo(540ex). Based on the same assumption also Fv(540ex) can be deconvoluted. In this case, the difference signal between the O-I_1 normalized 720ex and 540ex responses equals the Fv(I) contained in Fv(540ex). The Fv(II) component is obtained by subtracting the Fv(I) component from Fv(540ex).

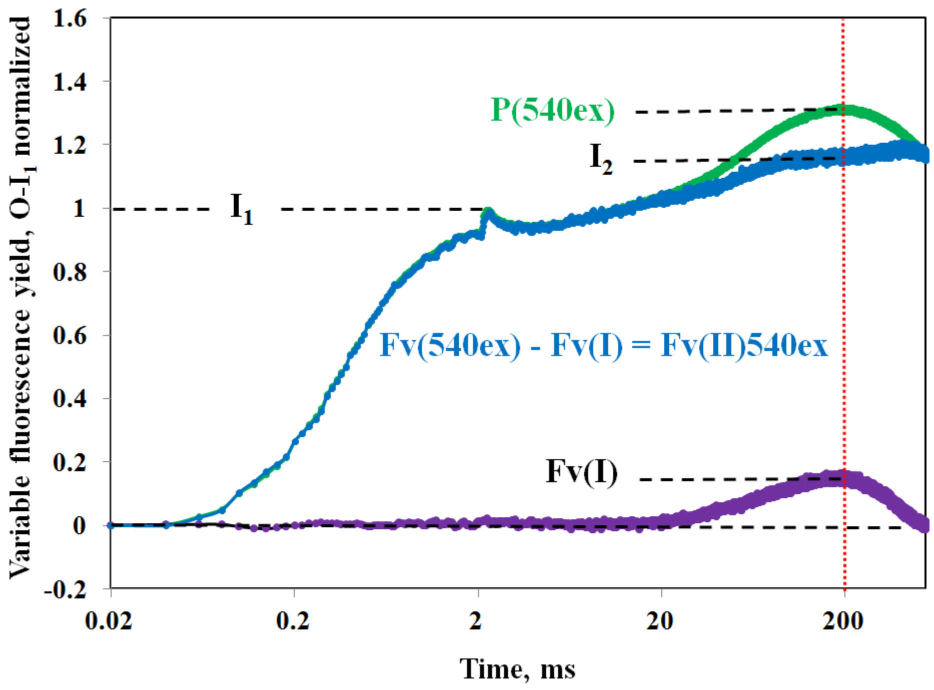

S10 Deconvolution of Fv(I) and Fv(II) in Fv(540ex). Dark-adapted *Chlorella*, with PQ-pool partially pre-reduced. Based on the original data of Figs. 4-6 (main text). Definition of the I_2-level so that at the Fv(I) peak (200ms, vertical red dotted line) I_2 = P(540ex) – Fv(I).

(6) Schreiber and Klughammer (2021) Fig. 8 revisited: Deconvolution of F(I) and F(II) based on the “extra Fv(I)” measured with F>700 compared with F<710.

First evidence for Fv(I) *in vivo* was obtained by Schreiber and Klughammer (2021) by comparative measurements of F>700nm (F>700) and F<710nm (F<710) in dilute suspensions of *Chlorella* upon excitation with 440nm pulse-modulated ML. In this case, after O-I_1 equalization the I_2-P transient was somewhat more pronounced in Fv(>700) compared to Fv(<710) and the “extra I_2-P” in Fv(>700) was considered to reflect the “extra Fv(I)” in Fv(>700). For deconvolution of F(I) and F(II) contained in the overall F>700 response, the whole Fv(I)>700 must be known, i.e. information is required on the factor y by which the “extra Fv(I)” has to be multiplied to obtain Fv(I)>700. In Fig. 8 of Schreiber and Klughammer (2021) determination of y was based on the assumption that the whole I_2-P transient is due to Fv(I). In this case, y corresponds to the factor by which the difference curve has to be multiplied to empirically match the O-I_1 equalized I_2-P(>700) kinetics. A factor of y = 1.5 empirically was found appropriate.

In analogy to the above considerations in Supplementary Materials section (4) on the F(I)/F(II) excitation ratio derived from F(720ex) and F(540ex) measurements, the relationship between the factor y and the Fo(I)/Fo(II) excitation ratio is also of interest for an assessment of the data in Fig. 8 of Schreiber and Klughammer (2021). As in this case fluorescence was excited with 440nm ML, the Fo values could be reliably corrected for minor background signals and, hence, determined with high accuracy. Based on the following values, definitions and equations the function y = 1840/(1840 – 208/z) can be derived, where z is the fraction of Fo(I) in Fo>700:

(1) Fo(<710) = 1632 = Fo(I)<710 + Fo(II)<710
(2) Fo(I)<710 = x*1632   Fo(II)<710 = (1-x)*1632
(3) Fo(>700) = 1840 = Fo(I)>700 + Fo(II)>700
(4) Fo(I)>700 = z*1840   Fo(II)>700 = (1-z)*1840
(5) Fo(II)<710 = Fo(II)>700 (valid after O-I_1 equalization)
(6) (1-x)*1632 = (1-z)*1840   x = z*1.1275 – 0.1275
(7) Fo(I)<710 = x*1632 = (z*1.1275 – 0.1275)*1632
(8) y = Fo(I)>700/Fo(I)<710
(9) y = z*1840/(z*1.1275 -0.1275)*1632 = 1840/(1840 – 208/z)

For z = 0.35, i.e. assuming that Fo>700 contains 35% Fo(I), a value of y = 1.48 is obtained, i.e. essentially the same value as the “I_2-P equalization factor” determined empirically in Schreiber and

Klughammer (2021). This means that Fo(I)/Fo(II) equals Fv(I)/Fv(II), under the assumption that the whole I_2-P transient is due to Fv(I), which argues in favor of this assumption.

The value of $y = 1.48$ means that after O-I_1 equalization in *Chlorella* the Fo(I) contained in F>700 exceeds the Fo(I) contained in F<710 just by a factor of 1.48. On first sight, relying on the wide-spread notion that F<710 consists almost exclusively of F(II), this factor seems “unlikely”. It becomes understandable, however, when recent insights on the association of LHCII with PSI-LHCI are taken into account. This aspect is dealt with in the main text, Discussion, section “Apparently “too large” contribution of F(I) to F<710 in Schreiber and Klughammer 2021”.
